# Licochalcone A prevents cognitive decline in a lipopolysaccharide-induced neuroinflammation mice model

**DOI:** 10.1186/s10020-025-01106-8

**Published:** 2025-02-11

**Authors:** Marina Carrasco, Laura Guzman, Jordi Olloquequi, Amanda Cano, Ana Fortuna, Manuel Vazquez-Carrera, Ester Verdaguer, Carme Auladell, Miren Ettcheto, Antoni Camins

**Affiliations:** 1 Department of Pharmacology, Toxicology and Therapeutic Chemistry, Faculty of Pharmacy and Food Science, Universitat de Barcelona, 08028 Barcelona, Spain; 2 Biomedical Research Networking Center in Neurodegenerative Diseases (CIBERNED), Instituto de Salud Carlos III, Madrid, Spain; 3 Institute of Neuroscience, Universitat de Barcelona, Barcelona, Spain; 4 Institut d’Investigació Sanitària Pere Virgili (IISPV), Reus, Spain; 5 Department of Biochemistry and Physiology, Faculty of Pharmacy and Food Science, Universitat de Barcelona, 08028 Barcelona, Spain; 6 Institute of Biomedical Sciences, Faculty of Health Sciences, Universidad Autónoma de Chile, Talca, Chile; 7 Ace Alzheimer Center Barcelona, Universitat Internacional de Catalunya, Barcelona, Spain; 8 Laboratory of Pharmacology and Pharmaceutical Care, Faculty of Pharmacy, University of Coimbra, 3000-548 Coimbra, Portugal; 9 Coimbra Institute for Biomedical Imaging and Translational Research, CIBIT/ICNAS, University of Coimbra, 3000-548 Coimbra, Portugal; 10 Networking Research Centre of Diabetes and Associated Metabolic Diseases (CIBERDEM), Instituto de Salud Carlos III, 28031 Madrid, Spain; 11 Institute of Biomedicine of the Universitat de Barcelona (IBUB), University of Barcelona, 08028 Barcelona, Spain; 12 Pediatric Research Institute-Hospital Sant Joan de Déu, 08950 Esplugues de Llobregat, Spain; 13 Department of Cellular Biology, Physiology and Immunology, Faculty of Biology, Universitat de Barcelona, 08028 Barcelona, Spain

**Keywords:** Cognitive decline, Neurodegeneration, Inflammation, Metabolism, Oxidative stress, Dendritic spines, Chalcone, Licorice

## Abstract

Inflammation plays a key role in the development of neurodegenerative disorders that are currently incurable. Licochalcone A (LCA) has been described as an emerging anti-inflammatory drug with multiple therapeutical properties that could potentially prevent neurodegeneration. However, its neuroprotective mechanism remains unclear. Here, we investigated if LCA prevents cognitive decline induced by Lipopolysaccharide (LPS) and elucidated its potential benefits. For that, 8-week-old C57BL6/J male mice were intraperitonially (i.p.) treated with saline solution or LCA (15 mg/kg/day, 3 times per week) for two weeks. The last day, a single i.p injection of LPS (1 mg/kg) or saline solution was administered 24 h before sacrifice. The results revealed a significant reduction in mRNA expression in genes involved in oxidative stress (S*od1, Cat, Pkm, Pdha1, Ndyfv1, Uqcrb1, Cycs* and *Cox4i1),* metabolism* (Slc2a1, Slc2a2, Prkaa1* and *Gsk3b)* and synapsis (*Bdnf, Nrxn3* and *Nlgn2)* in LPS group compared to saline. These findings were linked to memory impairment and depressive-like behavior observed in this group. Interestingly, LCA protected against LPS alterations through its anti-inflammatory effect, reducing gliosis and regulating M1/M2 markers. Moreover, LCA-treated animals showed a significant improvement of antioxidant mechanisms, such as citrate synthase activity and SOD2. Additionally, LCA demonstrated protection against metabolic disturbances, downregulating GLUT4 and P-AKT, and enhanced the expression of synaptic-related proteins (P-CREB, BDNF, PSD95, DBN1 and NLG3), leading all together to dendritic spine preservation. In conclusion, our results demonstrate that LCA treatment prevents LPS-induced cognitive decline by reducing inflammation, enhancing the antioxidant response, protecting against metabolic disruptions and improving synapsis related mechanisms.

## Introduction

It is well known that systemic inflammation is involved in neuropsychiatric pathologies including depressive-like behavior, and cognitive decline-related disorders such as Alzheimer's disease (AD) (Cunningham and Sanderson 2008). Peripheral activation of the immune system acutely activates innate immune cells, affecting a variety of tissues and organ systems (Gofton and Bryan 2012). Specifically, immune stimulation has been shown to influence neuroinflammatory response in the central nervous system (CNS) including changes in cytokine expression and alterations in cell-specific transcriptional programming, particularly in microglia. Consequently, brain function is compromised (Thomson et al. 2014 Apr; Smith et al. 2014; Prinz and Priller 2017). Lipopolysaccharide (LPS) is an endotoxin which constitutes one of the main components of Gram-negative bacteria membrane (Wang 2010). In preclinical studies, it is widely used as an stimulus to induce peripheral inflammation because it has been shown to activate toll-like receptor 4 (TLR4), initiating intracellular signaling pathways that involve Nuclear factor kappa-light-chain-enhancer of activated B cells (NF-κβ) and mitogen-activated protein kinases (MAPK), leading to upregulation of proinflammatory cytokines such as Interleukin-1 beta (IL-1β), Interleukin-6 (IL-6) and Tumor Necrosis Factor Alpha (TNF-α), enhancing blood brain barrier (BBB) permeability and leading to glial activation (Qin et al. 2007 Apr 1; Sanfeliu et al. 2023).

Moreover, it is known that neuroinflammation is accompanied by oxidative stress activation when the production of free radicals exceeds the antioxidant capacity of CNS. Specifically, reactive oxygen species (ROS) increase the vulnerability to brain cell damage and functional decline. In this context, ROS accumulation promotes the release of pro-inflammatory molecules, which, in turn, enhances homeostatic imbalance, thereby promoting the stimulation of ROS production. Additionally, LPS administration has been reported to induce metabolic alterations that reduce cerebral glucose uptake (Semmler et al. 2008 ) and carbohydrate metabolism, aggravating cognitive dysfunction (Takayuki Irahara et al. 2018 ).

As a result, several molecular pathways are altered simultaneously, contributing to loss of synapses, altered synaptic plasticity, disruption of brain energy metabolism, and increased neuronal death, ultimately, promoting cognitive decline and other behavioral disorders (Gouveia et al. 2024; Chunchai et al. 2024; Zhao et al. 2024; Gong et al. 2023; Sumire Matsuura et al. 2023; Gouveia et al. 2024). These facts evidence the complexity of neurodegenerative disorders.

However, the mechanisms through which these processes are interconnected with each other and contribute to cognitive decline are still unclear. Therefore, multitarget compounds could be considered as a potential effective treatment to counteract the alterations induced by inflammation (Chen et al. 2020; Neuroprotection 2021; Solleiro-Villavicencio and Rivas-Arancibia 2018; Medzhitov and Janeway).

Natural anti-inflammatory products, such as Licochalcone A (LCA), a phenolic compound found in licorice, have gained particular interest for their multi-target activity including potential anti-inflammatory and antioxidant properties, among others (Sarkar et al. 2022; Deng et al. 2023; Wang et al. 2021; AlDehlawi and Jazzar 2023; Maria Pia et al. 2019). Several studies have demonstrated its beneficial effects in pathologies where inflammation and oxidative stress play a relevant role, such as type 2 diabetes mellitus (Wang et al. 2024; Luo et al. 2021). Recently, LCA has received special attention in the field of neuroscience, not only because it has shown to cross the BBB (Lee et al. 2018; Yating et al. 2021), but also owing to its ability to reduce inflammatory response (Bhatia et al. 2023; Huang et al. 2017; Li et al. 2021 ), reenforcing that LCA may alleviate inflammation-related brain pathologies.

The present study aims to evaluate whether LCA protects from LPS-induced brain damage, leading to cognitive decline and associated pathological features. The results obtained in this study with mice pretreated with LCA prior to LPS administration supported the capacity of this phenolic compound to prevent brain damage and cognitive decline. Demonstrating that LCA would have a great therapeutic capacity to treat neurological diseases associated with dementia, such as AD, where inflammation plays an important role.

## Materials and methods

### Animals

For this study, six-week-old C57BL6/J male mice were used. All animals were given access to food or water ad libitum and kept under controlled temperature, humidity and Standard 12 h light–dark cycle conditions following the ethical guidelines defined by the European Committee (European Communities Council Directive 2010/63/EU). Every effort was made to reduce the number of animals and minimize animal suffering, in accordance with the manipulation protocol number 267/22, accepted by the ethics committee from the University of Barcelona. All the experiments were performed in accordance with the European Community Council Directive 86/609/EEC and the procedures established by the Departament d’Agricultura, Ramaderia i Pesca of the Generalitat de Catalunya.

### Treatment

**Fig. 1:**
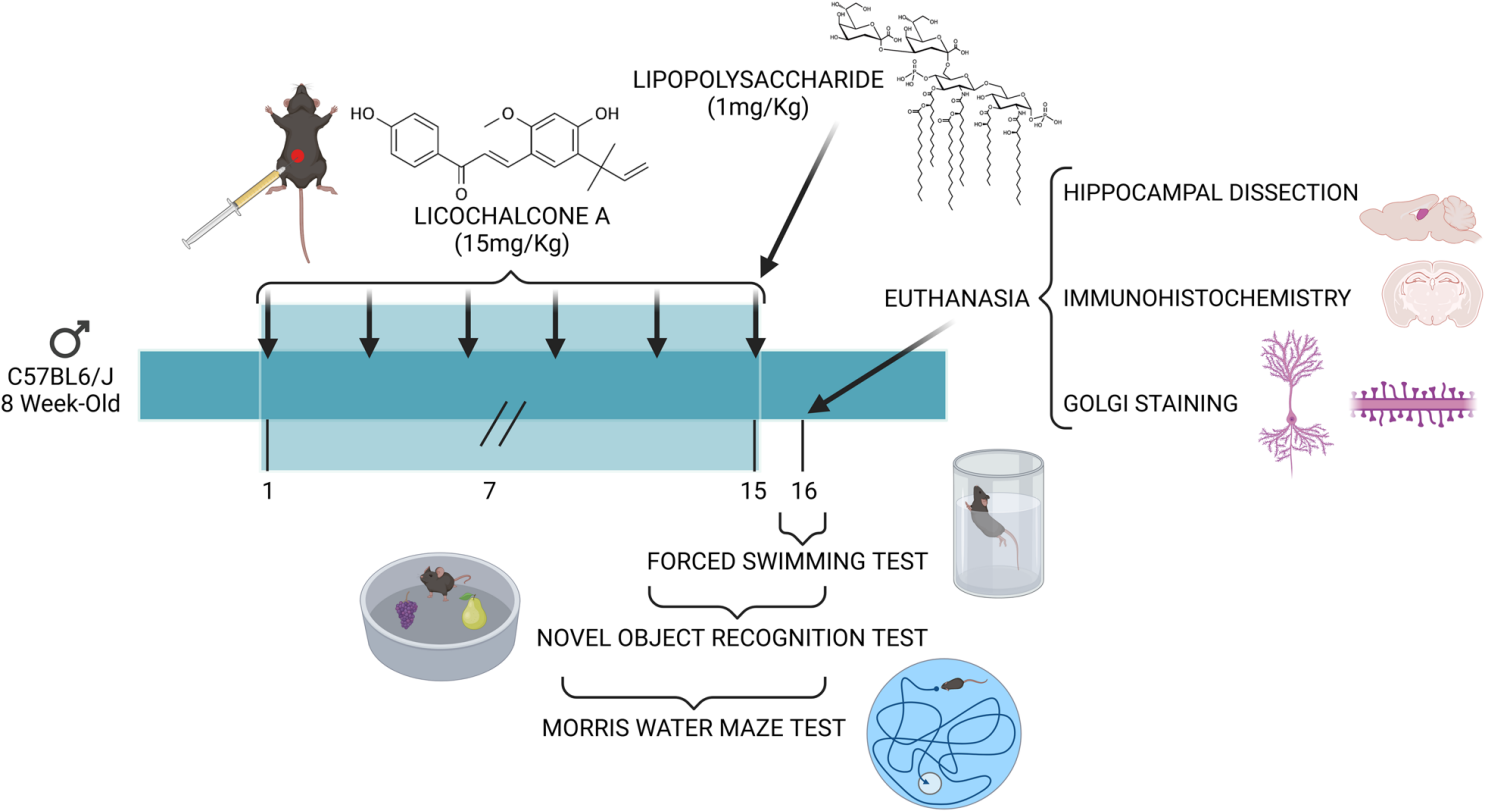
Graphical representation of experimental design. 8-week-old male C57BL6/J were treated i.p with 15 mg/kg of LCA, three times per week for 2 weeks. Finally, mice were exposed to a single i.p. injection of 1 mg/kg of LPS. Then, animals were subjected to three different behavioral tests: MWM (for 7 days), NORT (for 5 days) or FST (for 1 day). Finally, animals were sacrificed by cervical dislocation in order to obtain hippocampal samples and perform Golgi Staining, or by intracardially perfusion for immunochemistry. Image created with Biorender.com, agreement number WH274HDN5F

Mice were intraperitonially (i.p.) treated with saline solution (0.9% NaCl diluted in Water) or LCA at a dose of 15 mg/kg/day 3 times per week for 2 weeks. The last day, a single i.p. injection of 1 mg/kg LPS (L2880, Escherichia coli (O55:B5), Sigma-Aldrich, St. Louis, MO, USA) or saline solution i.p was administered and, 24 h later, animals were sacrificed by cervical dislocation for hippocampi isolation and dendritic spine staining or by intracardial perfusion with 4% (v/v) paraformaldehyde for inmunohistochemical analyses. Animals were divided into 4 groups: “Saline”, “LCA”, “LPS” and “LCA + LPS”. A graphical representation of the experimental design is depicted in Fig. 1.

### Behavioral tests

Just before euthanasia, all the animals were subjected to behavioral tests in order to study long-term memory and depression-like behavior.

#### Morris water maze

Mice were subjected to the Morris Water Maze (MWM) test to assess the spatial long-term memory and learning abilities. The procedure was performed in a circular pool of 100 cm diameter, divided into four quadrants with a scape platform hidden 1 cm under the water, previously stained with liquid latex. Four different spatial cues were placed around the pool to foster spatial orientation and kept constant throughout the experiment. Water temperature and luminosity were maintained at 28ºC and 30Lux, respectively, during the entire procedure.

This test was divided into two different phases: training and probe test. During the training period, the animals were introduced to the pool from 5 different locations, for 6 consecutive days and were allowed to swim freely to locate the hidden platform for 1 min. If the animal was unable to reach the platform, it was placed on it for 30 s.

On the probe test, the 7th day, the platform was removed, and the animal was introduced into the pool from a single position, allowing it to swim freely for 1 min. Acquired data were analyzed using SMART V3.0 (Panlab Harvard Apparatus, Germany) video tracking system, with results calculated individually for each animal.

#### Novel object recognition test

The non-spatial recognition memory was evaluated by the Novel Object Recognition Test (NORT). The procedure was performed in a circular open-field box with a diameter of 40 cm, under constant 30 lx illumination. The test was divided into three phases: habituation, familiarization, and test. During the habituation phase, each mouse was placed in the arena without objects for three consecutive days, for 10 min each session. The familiarization phase was conducted on the fourth day; during this period, each mouse was allowed to explore two identical objects (A and A’) placed in the middle of the arena for 10 min. Finally, the test phase was conducted on the fifth day, where each mouse was exposed to a familiar object (A) and a novel object (B) for 10 min.

After each trial, the objects and the open-field box were cleaned with 70% ethanol to avoid olfactory distractions. Every trial was recorded, and the Discrimination Index (DI) was determined using the following equation:

$$DI=\frac{Time\, exploring\, B-Time\, exploring A}{Time\, exploring A+Time\, exploring\, B}$$

Exploration was defined as looking, sniffing or touching an object. Mice with a total exploration time < 5 s were removed from the analyses.

#### Forced swimming test

Depressive-like behavior was evaluated through the Forced Swimming Test (FST). To carry out this behavioral test, mice were submerged in a beaker (20 × 30 cm) filled with water for 6 min. The experiment was recorded, and the percentage of immobility was analyzed during the last four minutes, as many animals are very active during the first minutes of the test (Yankelevitch-Yahav et al. 2015). Immobility was considered as the absence of any movement except for those necessary to keep the nose above the water (Yankelevitch-Yahav et al. 2015). Throughout the experiment, the water temperature was kept constant at 26–28ºC, and the water level was maintained to prevent the animal from touching the bottom or jumping out.

### mRNA isolation

After hippocampal dissection, the tissue was kept at −80ºC until use. mRNA extraction was performed on hippocampal samples by homogenizing the tissue with TRIsure™ (BIO-38033; Bio line GmbH, London, UK). The homogenates were centrifugated at 12.000 g for 5 min at 4 ºC. The supernatants were collected and transferred to new tubes where chloroform was added. After another centrifugation cycle, the upper layer was transferred to another tube, and isopropanol was added to the solution, allowing it to rest on ice for 10 min. Subsequently, the samples were centrifuged for 10 min at 14.000* g* at 4 °C. The obtained pellet was washed with 70% (v/v) ethanol and centrifugated again at 7.500 g for 5 min at 4ºC. Finally, the remaining pellet was left to dry and diluted in diethylpyrocarbonate (DEPC)-treated water.

RNA concentration and integrity were analyzed with a NanoDrop™ One/OneC Microvolume UV–Vis Spectrophotometer (Thermo Scientific, Waltham, Massachusetts, United States). Finally, 2.000 ng of mRNA per sample were converted to cDNA by reverse transcription with a High-Capacity Reverse transcription Kit (4,368,813; Applied Biosystems, Foster City, CA, USA).

### TaqMan array

TaqMan® Array 96 -Well FAST plate (ThermoFisher Scientific, Inc.) was used to analyze a total of 48 genes related to oxidative stress, metabolism, and synapsis. The sequences corresponding to *18S, Actb, Gapdh, Hprt* and *Gusb* were tested as housekeeping genes in all samples. Since *Actb* showed least variability, it was selected to perform the analysis. The targets included in the Array were: *Slc2a1, Slc2a2, Slc2a3, Slc2a4, Insr, Irs2, Prkaa, Akt1, Akt2, Creb1, Gsk3β, Pparγ, Pparγc1α, Ptpn1, Hk1, Hk2, Pfkp, Pkm, Pdha1, Pdha2, Ndufv1, Sdha, Sdhb, Uqcrc1, Uqcrb, Cycs, Cox4i1, Atp5b, Sod1, Gpx1, Cat, Bdnf, Ntrk2, Ppp1r9b, Syp, Dlg4, Nrxn1, Nrxn2, Nrxn3, Nlgn1, Nlgn2, Nlgn3*. No data was reported of *Pparγ* and *Pdha2* since the TaqMan® probes produced either a CT value over 35. Specific descriptions for each of the genes included in the study can be found in Table 1.

**Table 1:** Specific descriptions of the genes included in the TaqMan® array

Gene	Full Protein Name
SLC2A1	Glucose Transporter Type 1 (GLUT1)
SLC2A2	Glucose Transporter Type 2 (GLUT2)
SLC2A3	Glucose Transporter Type 3 (GLUT3)
SLC2A4	Glucose Transporter Type 4 (GLUT4)
INSR	Insulin Receptor (IR)
IRS1	Insulin Receptor Substrate 1 (IRS1)
IRS2	Insulin Receptor Substrate 2 (IRS2)
PRKAA1	Protein Kinase AMP-Activated Catalytic Subunit Alpha 1 (AMPK)
AKT1	Protein Kinase B (AKT) 1
AKT2	Protein Kinase B (AKT) 2
CREB1	CAMP Responsive Element Binding Protein 1 (CREB1)
GSK3Β	Glycogen Synthase Kinase 3 Beta (GSK3β)
PPARΓ	Peroxisome proliferator-activated receptor gamma (PPARγ)
PPARΓC1Α	Peroxisome proliferator-activated receptor gamma coactivator 1-alpha (PGC1α)
PTPN1	Protein tyrosine phosphatase 1B (PTP1B)
HK1	Hexokinase 1 (HK1)
HK2	Hexokinase 2 (HK2)
PFKP	Phosphofructokinase (PFK)
PKM	Pyruvate kinase (PK)
PDHA1	Pyruvate dehydrogenase subunit 1 (PDHA1)
PDHA2	Pyruvate dehydrogenase subunit 2 (PDHA2)
NDUFV1	NADH dehydrogenase ubiquinone flavoprotein 1 (NDUFV1; subunit of OXPHOS CI)
SDHA	Succinate Dehydrogenase Complex Flavoprotein Subunit A (SDHA; subunit of OXPHOS CII)
SDHB	Succinate Dehydrogenase Complex Flavoprotein Subunit B (SDHB; subunit of OXPHOS CII)
UQCRC1	Ubiquinol-Cytochrome C Reductase Core Protein 1 (UQCRC1; subunit of OXPHOS CIII)
UQCRB	Ubiquinol-cytochrome c reductase binding protein (UQCRB; subunit of OXPHOS CIII)
CYCS	Cytochrome C (CYC)
COX4I1	Cytochrome C Oxidase Subunit 4I1 (COX4i1; subunit of OXPHOS CIV)
ATP5B	ATP synthase F1 subunit beta (ATP5B; subunit of OXPHOS CV)
SOD1	Superoxide dismutase 1 (SOD1)
GPX1	Glutathione peroxidase 1 (GPX1)
CAT	Catalase (CAT)
BDNF	Brain-Derived Neurotrophic Factor (BDNF)
NTRK2	Neurotrophic Receptor Tyrosine Kinase 2 (BDNFR)
PPP1R9B	Protein Phosphatase 1 Regulatory Subunit 9B – Neurabin2 (NRBN)
SYP	Synaptophysin (SYP)
DLG4	Postsynaptic density protein 95 (PSD95)
NRXN1	Neurexin 1 (NRXN1)
NRXN2	Neurexin 2 (NRXN2)
NRXN3	Neurexin 3 (NRXN3)
NLGN1	Neuroligin 1 (NLGN1)
NLGN2	Neuroligin 2 (NLGN2)
NLGN3	Neuroligin 3 (NLGN3)

During the procedure, 80 ng of cDNA and equal volume of Master Mix were mixed to a final volume of 10 µL per well. Each plate was immediately run on a Step One Plus real-time PCR System (Life Technologies, Grand Island, NY, USA) under the following parameters: 1 cycle of 2 min at 50º, 1 cycle of 20 s at 95ºC and 40 cycles of 1 s at 95ºC followed by 20 s at 60ºC.

### Real time polymerase chain reaction

After obtaining cDNA from RNA, as previously described, the equivalent cDNA amount was analyzed in duplicate for each gene. For this, SYBR Green with ROX (Thermo Scientific Maxima SYBR Green qPCR Master Mix (2X); K0253; Thermo Scientific) was mixed with an equal volume of cDNA and the corresponding forward and reverse primer sequences, detailed in Table 2. The plates were run on a Step One Plus real-time PCR system (Life Technologies, Grand Island, NY, USA), and the results were normalized to the Gapdh and expressed relative to the Saline group, in order to evaluate gene expression variations.

**Table 2:** Primers for RT-PCR

Gene	Forward primer	Reverse primer
TREM2	CTGGAACCGTCACCATCACT	CACCCTCGAAACTCGATGAC
TL4R	AGGCAGCAGGTGGAATTGTATC	TCGAGGCTTTTCCATCCAATAG
CD86	ACGATGGACCCCAGATGCACCA	GCGTCTCCACGGAAACAGCA
ARG1	CTTGCGAGACGTAGACCCTG	TCCATCACCTTGCCAATCCC

### Protein extraction

Frozen hippocampi were homogenized in lysis buffer (Tris HCl 1 M pH 7.4, NaCl 5 M, EDTA 0.5 M pH 8, Triton, distilled H20) containing protease and phosphatase inhibitor cocktails (Complete Mini, EDTA-free; Protease Inhibitor cocktail tablets), kept on ice for 30 min and centrifuged at 14.000 g for 10 min at 4 ºC. Total protein concentration was determined with Pierce^™^ BCA Protein Assay Kit (#23,225, Thermo Scientific^™^, Rockford, USA).

### Western blotting

10 µg protein samples were denatured in 2 × Sample Buffer (0.25 M Tris pH 6.8, 4% (w/v) SDS, 200 mM dithiothreitol (DTT), 20% (v/v) glycerol and bromophenol blue) at 95 °C for 5 min. Afterwards, samples were loaded into a 10% (v/v) SDS–polyacrylamide gels and separated on a SDS-PAGE gel at 120 V for 90 min. Subsequently, proteins were transferred to an activated polyvinylidene fluoride membrane for 2 h at 200 mA. The resulting membrane was blocked for 1 h at room temperature (RT) with a 5% (w/v) bovine serum albumin (BSA) solution, in Tris-buffered saline (TBS) (150 mM NaCl, 25 mM Tris–HCl pH 7.6) with 0.1% (v/v) Tween20 (TBS-T). Membranes were incubated at 4 °C overnight (O/N) with primary antibodies, detailed in Table 3.

**Table 3:** Antibodies for western blotting and inmnunohistochemistry

Protein	Antibody
SOD2	MA5-31,514 (Invitrogen)
PERK	3192 (Cell Signaling Technology)
P-PERK Thr980	3179 (Cell Signaling Technology)
GLUT4	SC-7938 (Santa Cruz Biotechnology)
AKT	9272 (Cell Signaling Technology)
P-AKT Ser473	4060 (Cell Signaling Technology)
CREB	9197 (Cell Signaling Technology)
P-CREB Ser133	9198 (Cell Signaling Technology)
LIMK1	3842 (Cell Signaling Technology)
DBN1	ABN 207 (Merck Millipore)
PSD95	MAB1598 (Merck Millipore)
NEUROLIGIN 3	Ab186307 (Sigma)
BDNF	SC-546 (Santa Cruz Biotechnology)
GAPDH	MAB374 (Merck Millipore)
β-ACTIN	A5441 (Sigma)
TUBULIN	T4026 (Sigma)
GFAP	Z0334 (Dako)
IBA1	O19‑19,741 (Wako)
2nd‑ary Goat anti‑Rabbit	31,460 (Invitrogen
2nd‑ary Goat anti‑Mouse	31,430 (Invitrogen)
2nd‑ary Alexa Fluor 488 (Goat‑Anti Mouse)	A11001 (Life Technologies)
2nd‑ary Alexa Fluor 594 (Goat‑Anti Rabbit)	A11080 (Life Technologies)

Membranes were washed 3 times for 5 min with TBS-T, incubated for 1 h at RT with the corresponding secondary antibodies (Table 3) and washed 3 times for 5 min with TBS-T. The immunoreactive protein bands were visualized with Immobilon® Western Chemiluminescent HRP Substrate (#WBKLS0500, Merck Millipore, Darmstadt, Germany) using ImageQuant LAS 500 (GE Healthcare, Chicago, IL, USA).

Finally, membranes were washed and incubated with the primary antibody of the housekeeping protein for 1 h at RT, washed 3 times for 5 min, and incubated for 1 h at RT with the corresponding secondary antibody (Table 3). The immunoreactive protein bands were visualized by chemiluminescence with Pierce ECL Western Blotting Substrate (#32,106 Thermo Fisher Scientific, Waltham, Massachusetts, United States) using ImageQuant LAS 500 (GE Healthcare, Chicago, IL, USA). The optical density of the bands obtained was quantified with Image Lab Software (Bio-Rad, Hercules, California, United States).

### Inmunohistochemistry

After intracardial perfusion, brains were isolated and stored in 4% (v/v) PFA for 24 h at 4ºC. The following day, the solution was replaced with a new one made of 30% sucrose and 2% sodium azide diluted in 0.1 M phosphate buffered saline (PBS) for at least 3 days. Once the brains were dehydrated, they were frozen at −80ºC, and coronal sections of 20 μm of thickness were obtained using a cryostat (Leica Microsystems, Wetzlar, Germany). The sections were kept at −20ºC in a cryoprotectant solution (10% PB 0,1 M, 30% Ethylene glycol, 30% Glycerol) until use.

To perform immunohistochemistry techniques, free-floating sections were used. These slices were rinsed three times for 5 min in 0.1 M PBS (pH 7.35), then 5 times for 5 min in PBS 0.1 M containing 0.5% (v/v) Triton X-100 (PBS-T). Afterwards, they were incubated in a blocking solution (10% fetal bovine serum (FBS), 1% Triton X-100, PBS 0.1 M + 0.2% gelatin) for 2 h at RT. Subsequently, the sections were washed 5 times for 5 min with PBS-T and incubated O/N at 4 °C with the corresponding primary antibody, detailed in Table 3. The next day, brain slices were washed 6 times for 5 min with PBS-T and incubated with the corresponding secondary antibody (Table 3) for 2 h at RT, followed by three washes with PBS-T and washes with 0.1 M PBS for 5 min each. Additionally, nuclei were stained with 0.1 μg/mL Hoechst (Sigma-Aldrich, St Louis, MO, United States) for 8 min in the dark at RT and washed 3 times with 0.1 M PBS for five minutes.

Finally, slices were mounted on Superfrost® microscope slides using Fluoromount-G ™ medium (#00–4958-02, Invitrogen, California, USA). Image acquisition was obtained using an epifluorescence microscope (BX61 Laboratory Microscope, Melville, NY OlympusAmerica Inc.) and quantified by ImageJ (Schindelin et al. 2012).

### Dendritic spine quantification

To quantify the dendritic spine density, fresh brains were removed from the skull and processed immediately following the protocol provided in the FD GolgiStainTM Kit (#PK401, FD Neurotechnologies, Inc, Columbia, USA). Afterwards, 63 × images were obtained with a Leica Thunder Microscope (Leica Thunder Imager; Leica Microsystems) and processed with ImageJ (Schindelin et al. 2012). The quantified dendrites were taken from the secondary branches and the terminal dendrites of dentate gyrus (DG), as well as the secondary branches of the cornu Ammonis 1 (CA1) basal zone and terminal dendrites in CA1 apical zone. When quantifying the secondary branches, the first 20 µm from the beginning of the ramification were excluded and the following 30 µm were analyzed. In contrast, when quantifying the terminal fragment, the last 20 µm of the final dendrite were excluded and the following 30 µm were quantified. The analysis was performed with 5 dendrites from each zone per animal, and the results were expressed as the number of spines in 30 μm of dendrite.

### Citrate synthase activity

Citrate synthase activity was detected on hippocampus homogenate as described in the protocol provided by the Citrate Synthase Assay Kit (#ab239712, Abcam, Cambridge, UK) and corrected with the protein quantification obtained with the Pierce™ BCA Protein Assay Kit (#23,225, Thermo Scientific™, Rockford, USA).

### Statistical analysis

Statistical analysis was performed using GraphPad Prism version 8.3.0 (San Diego, CA, United Stated, www.graphpad.com). When comparing two groups, an unpaired t-student test or Mann–Whitney test was performed, depending on whether the results passed the Shapiro–Wilk normality test or not, respectively. Meanwhile, when comparing the four groups a two-way ANOVA followed by Tukey’s post-test for multiple comparisons was applied. Differences were considered statistically significant when *p < 0.05, **p < 0.01, ***p < 0.001 and ****p < 0.0001.

## Results

### LPS induces alterations in the expression of genes related to oxidative stress, metabolism and synapsis

**Fig. 2:**
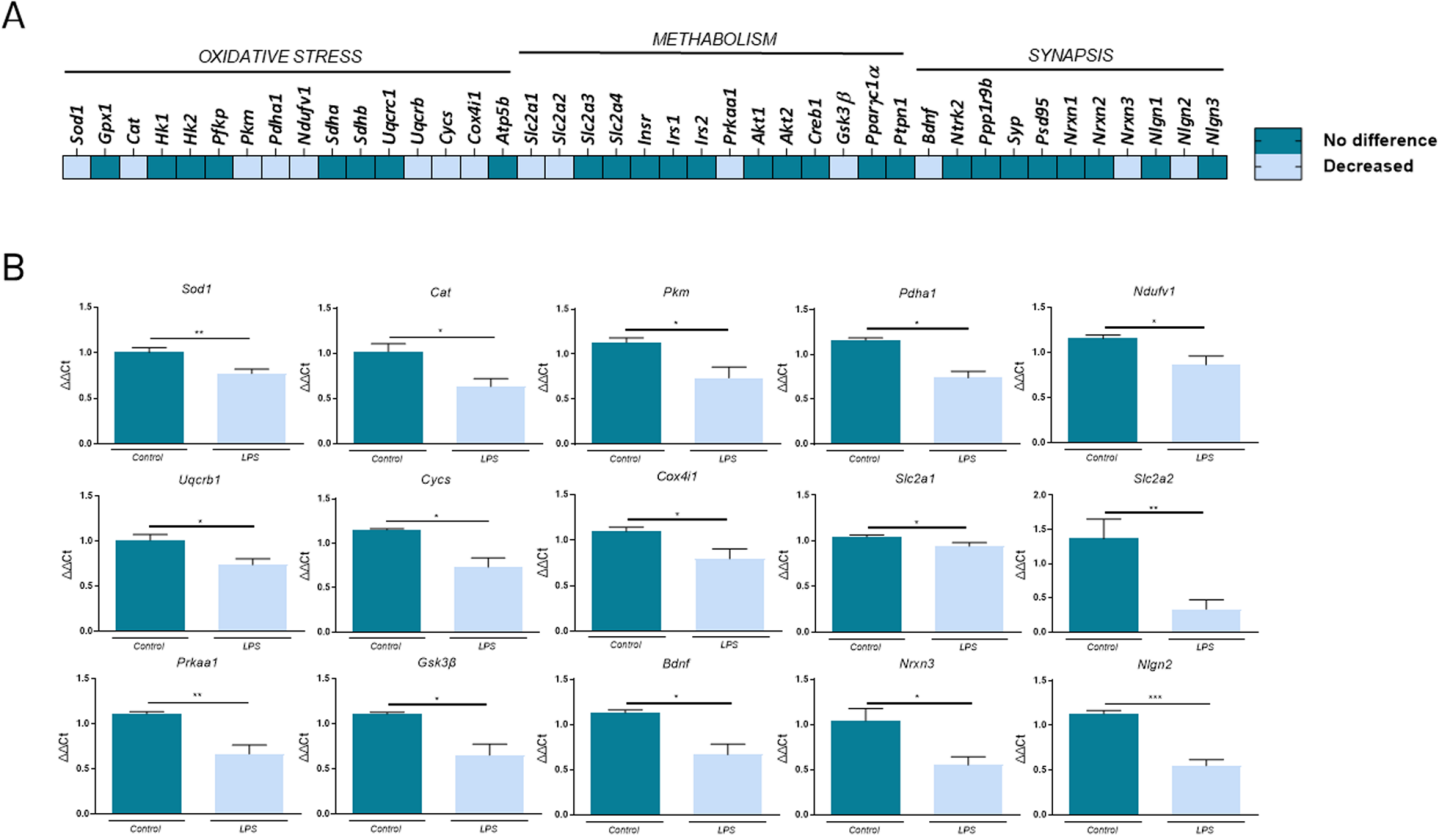
Heatmap TaqMan Array screening of 43 gene related to neurodegeneration (n = 5–8 independent mice per group). Representative histogram of ΔΔCt results, expressed as mean ± SEM. Statistical analysis was performed by t-student or Man Whitney test where * denotes p < 0.05, **P < 0,01 and *** p < 0.001

To characterize the brain damage in the well-known LPS-induced inflammation murine model and elucidate potential targets to prevent and modulate neuronal damage, it was performed a screening of 43 genes related to oxidative stress, metabolism and synapsis (Fig. 2), three key mechanisms related to cognitive dysfunction (McDonald et al. 2023 ; Domenico et al. 2017; Lee Mosley et al. 2006; Lin and Flint Beal 2006). Our results showed a significant reduction of genes involved in oxidative stress, including S*od1, Cat, Pkm, Pdha1, Ndyfv1, Uqcrb1, Cycs* and *Cox4i1*, in LPS mice versus control (** for *Sod1*: p < 0,01; for the rest: p < 0,05). Additionally, the expression of genes involved in metabolic processes was also significantly downregulated after LPS administration vs control, including *Slc2a1, Slc2a2, Prkaa1* and *Gsk3b* (*Slc2a1*: *p < 0,05; *Slc2a2*: **p < 0,01; *Prkaa1*: **p < 0,01 and *Gsk3β* *p < 0,05. Finally, synapsis-related genes, such as, *Bdnf, Nrxn3* and *Nlgn2* were significantly reduced in the LPS group compared to controls (p < 0,05 for *Bdnf* and *Nrxn3* and ***p < 0,001 for *Nlgn2*).

### LCA mitigates the neuroinflammation induced by lps pre-treatment

**Fig. 3:**
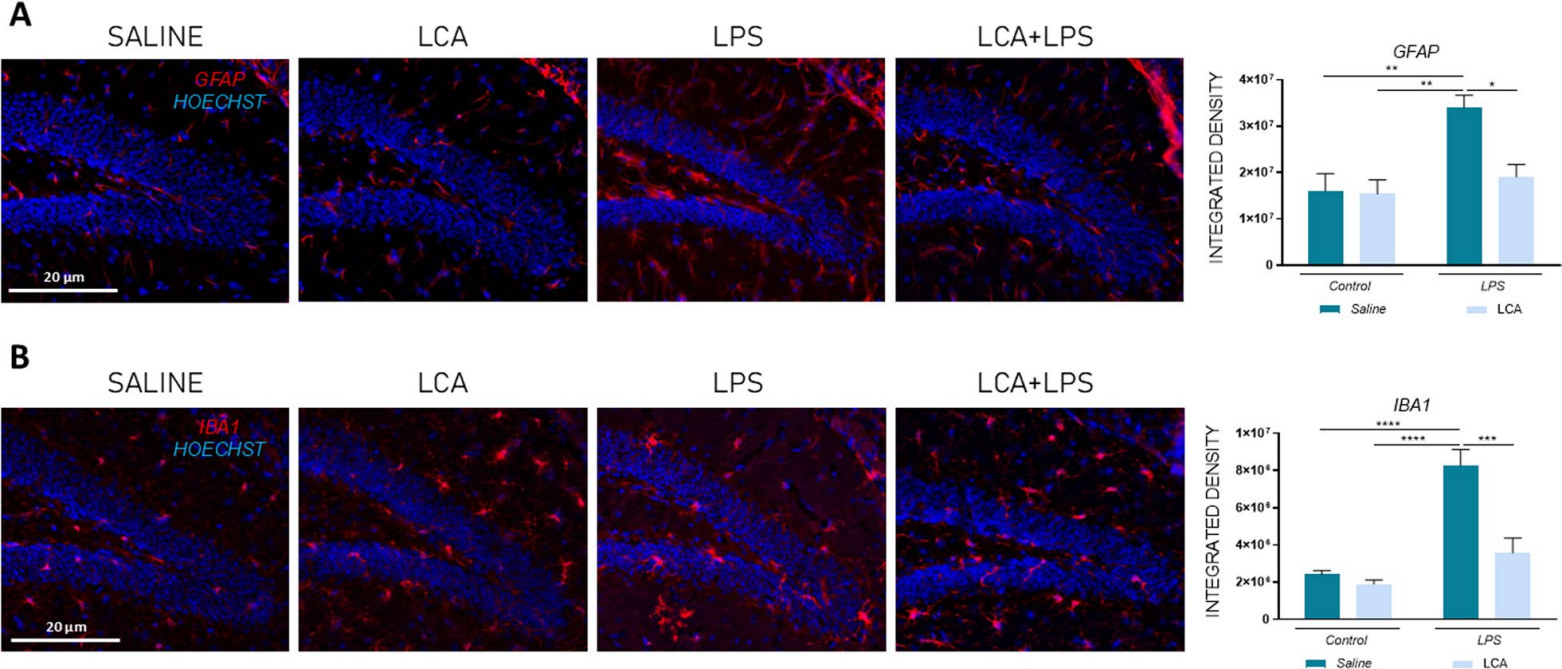
Immunofluorescence against GFAP (red), IBA1 (red) and Hoescht (blue) X20. Representative histogram of integrated density. N = 5 independent samples per group, with at least 5 slices analyzed per sample. Results are expressed as mean ± SEM. Statistical analysis was performed by two-way ANOVA and Tukey´s post-hoc where * denotes p < 0.05, ** p < 0.01, *** p < 0.001 and **** p < 0.0001

**Fig. 4:**
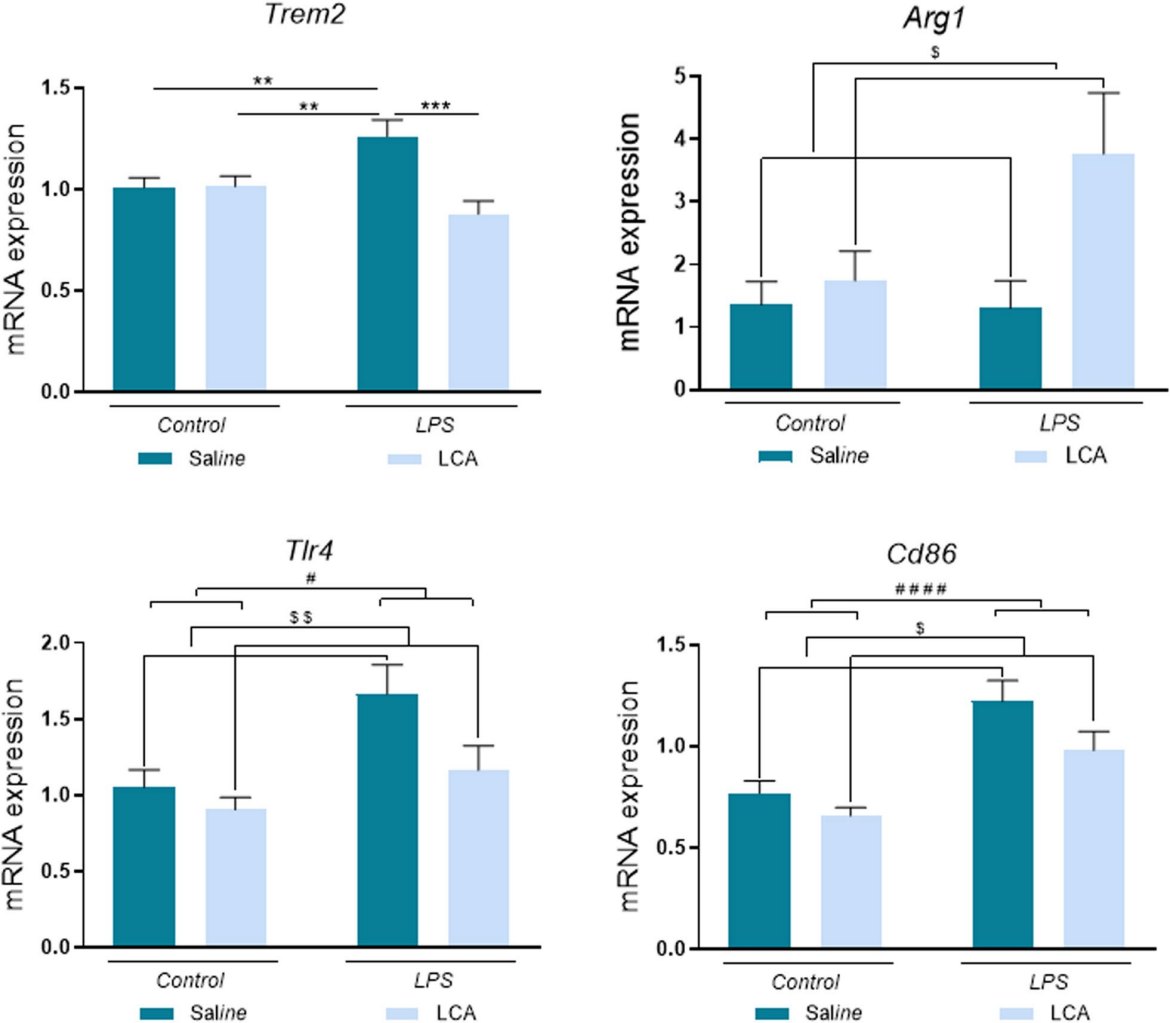
Representative histogram of mRNA expression levels. N = 5–8 independent samples per group. Results are expressed as mean ± SEM. Statistical analysis was performed by two-way ANOVA where $ denotes p < 0.05 and $$ p < 0,001 compared to non-LCA groups and Tukey´s post-hoc where * denotes p < 0.05, ** p < 0.01, *** p < 0.001 and **** p < 0.0001

Astrocytes and microglia reactive profiles were analyzed in the DG of the hippocampus through the detection of glial fibrillary acidic protein (GFAP) and ionized calcium-binding adapter molecule 1 (IBA1), respectively, as neuroinflammatory markers (Fig. 3). These data demonstrated a significant increase in astrogliosis and microgliosis after LPS exposure versus the control group (SAL vs LPS **p < 0,01 and **** < 0,0001 for GFAP and IBA1, respectively) that was significantly mitigated when the animals were previously treated with LCA (LPS vs LCA + LPS *p < 0,05 and ***p < 0,001 for GFAP and IBA1, respectively), demonstrating that LCA prevents glial reactivity induced by acute LPS exposure. In addition, the hippocampal expression levels of anti-inflammatory and pro-inflammatory genes were evaluated (Fig. 4). Specifically, the triggering receptor expressed on myeloid cells 2 (TREM2), which has been demonstrated to regulate inflammatory processes through microglia modulation (Sun et al. 2024 ), showed higher expression levels in the LPS group compared to saline. By contrast, when those animals were previously treated with LCA, *TREM2* expression levels were downregulated (SAL vs LPS **p < 0,01; LPS vs LCA + LPS ***p < 0,001).

Additionally, arginase 1 (ARG1), an anti-inflammatory gene (Li et al. 2022), showed a significant increase in mRNA expression after LCA treatment, independently of LPS exposure (non-LCA vs LCA $p < 0,05).

By contrast, mRNA expression of pro-inflammatory gens such as *Tlr4* and *Cd86* was significantly increased after a single dose of LPS compared to saline (non-LPS vs LPS *p < 0,05 and ****p < 0,0001 for *Tlr4* and *Cd86*, respectively). These expressions were significantly reduced in the animals previously treated with LCA (non-LCA vs LCA $$ p < 0,01 and $p < 0,05 for *Tlr4* and *Cd86*, respectively).

### LCA improves mithocondrial function and protects against oxidative stress after lps expousure

**Fig. 5:**
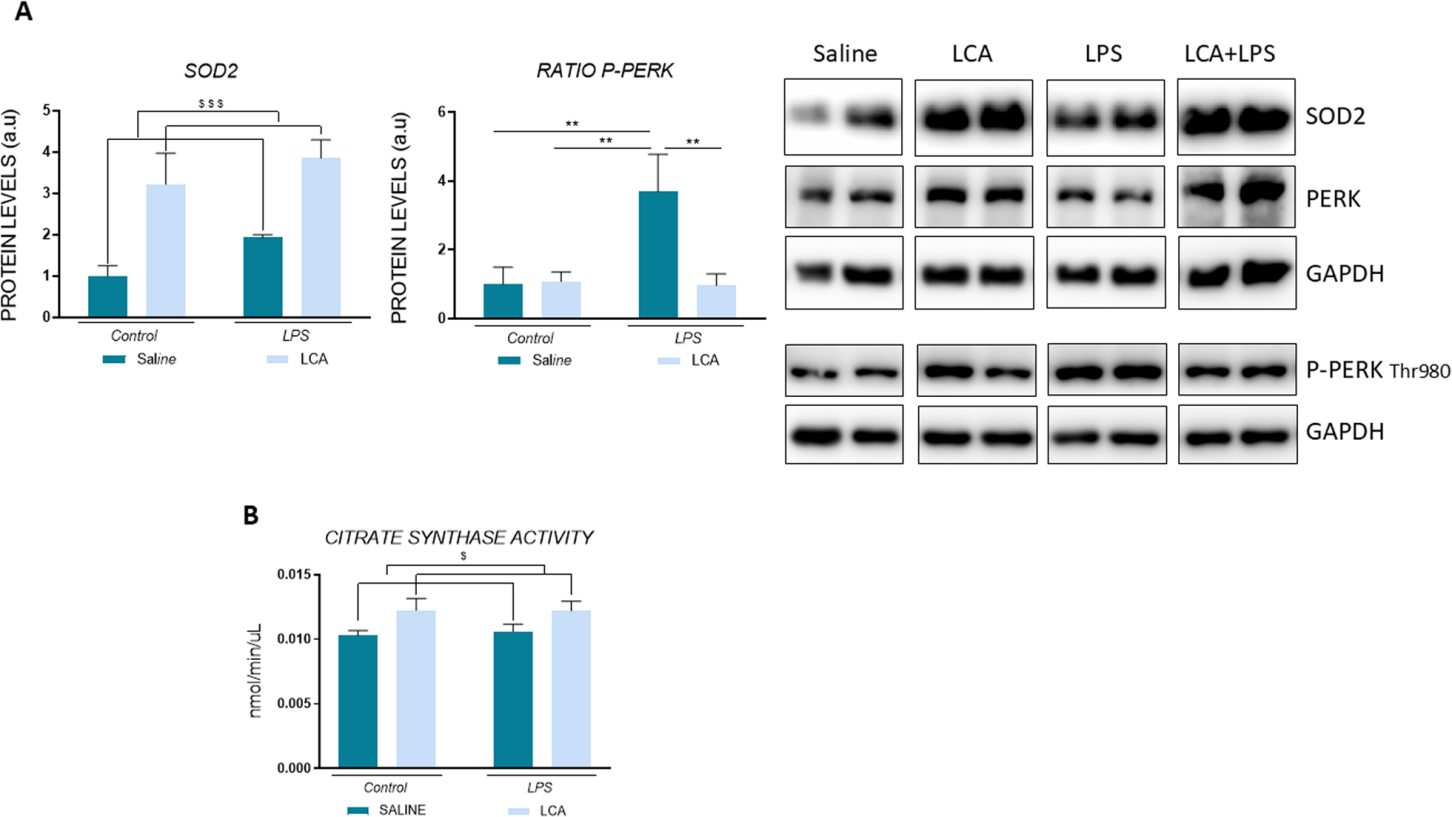
Representative histogram of protein expression levels. N = 3–4 independent samples per group. Results are expressed as mean ± SEM. Statistical analysis was performed by two-way ANOVA where $ denotes p < 0.05 and $$$ p < 0,0001 compared to non-LCA groups and Tukey´s post-hoc where ** denotes p < 0.01

Several studies have suggested oxidative stress and mitochondria function regulation as therapeutic targets for neuroprotection (Lee Mosley et al. 2006; Lin and Flint Beal 2006; Borsche et al. 2021; Flynn and Melovn 2013). Therefore, the determination of superoxide dismutase 2 (SOD2) protein levels, an antioxidant enzyme, was performed in the hippocampus of mice (Fig. 5). Results showed a significant higher level in LCA-treated animals, independent to genotype (non-LCA vs LCA $$$p < 0,001). In line with this, the enzymatic activity of citrate synthase was analyzed as a marker of mitochondrial function (Fig. 5). Our data showed a similar profile to SOD2, where animals treated with LCA demonstrated a significant increase in this enzyme (non-LCA vs LCA $p < 0,05). Moreover, the levels of phospho-protein kinase R-like endoplasmic reticulum kinase (PERK) that is responsible to endoplasmic reticulum (ER) stress and mitochondria dysfunction (Cullinan and Diehl 2004 ), showed a significant increase in LPS-exposed animals compared to saline, that was significantly prevented when these mice were previously treated with LCA (SAL vs LPS **p < 0,01; LPS vs LCA + LPS **p < 0,01) (Fig. 5).

### LCA prevents metabolic alterations induced by lps administration

**Fig. 6:**
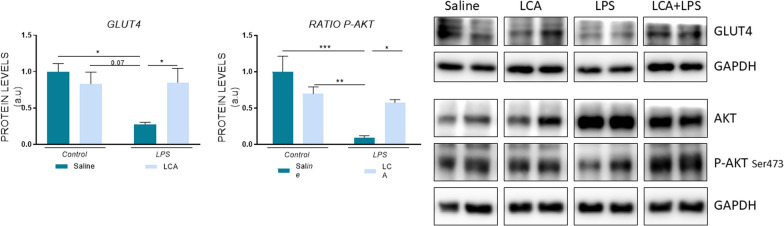
Representative histogram of protein expression levels. N = 3–4 independent samples per group. Results are expressed as mean ± SEM. Statistical analysis was performed by two-way ANOVA and Tukey´s post-hoc where * denotes p < 0.05, ** p < 0,01 and ***p < 0,001

It is well known that alterations in glucose metabolism play a crucial role in neurodegeneration (McDonald et al. 2023 ). Therefore, proteins related to insulin signaling pathway were evaluated in the hippocampus. The results obtained showed that a single dose of LPS induced metabolic alterations that could be prevented with a previous treatment of LCA (Fig. 6). Specifically, LPS-treated mice showed a significant reduction of glucose transporter type 4 (GLUT4) levels, a glucose transporter specific for the access of glucose into neurons in the hippocampus (Yonamine et al. 2023). By contrast, LCA significantly protected against this reduction (SAL vs LPS *p < 0,05; LPS vs LCA + LPS *p < 0,05).

Additionally, it has been reported that the activation of protein kinase B (AKT) at Ser473 regulates different biological functions such as metabolism and transcriptional regulation, promoting neuronal cell survival (Rai et al. 2019). The present data demonstrated a significant reduction in this phosphorylation in the LPS group compared to saline, which was prevented with previous treatment of LCA (SAL vs LPS ***p < 0,001; LPS vs LCA + LPS *p < 0,05).

### LCA enhances synaptic plasticity and dendritic spine preservation after lps administration

**Fig. 7:**
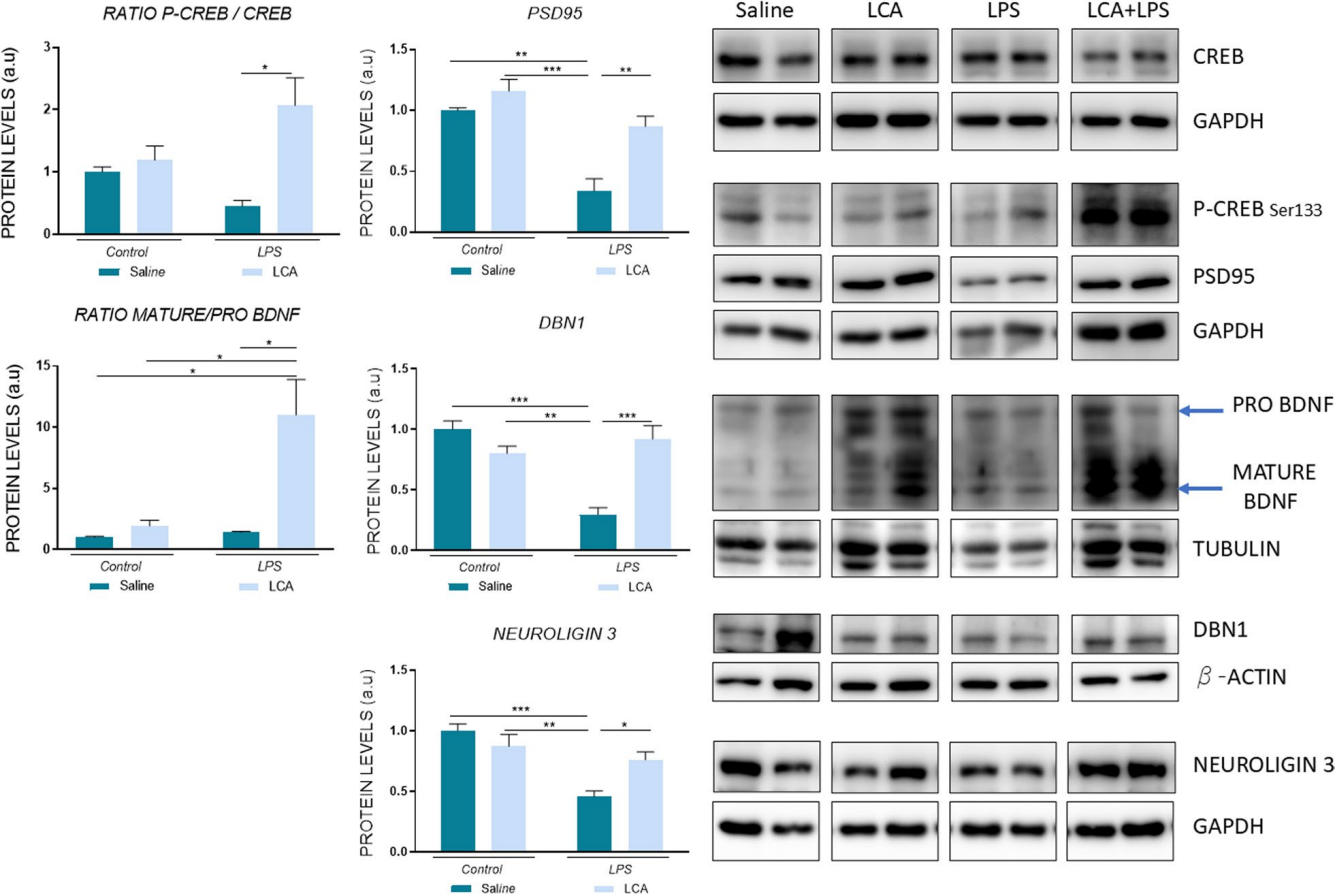
Representative histogram of protein expression levels. N = 3–4 independent samples per group. Results are expressed as mean ± SEM. Statistical analysis was performed by two-way ANOVA and Tukey´s post-hoc where * denotes p < 0.05, ** p < 0,01 and ***p < 0,001

Synaptic function depends on the structural integrity of the synapse, which is regulated by various mediators such as brain-derived neurotrophic factor (BDNF) and postsynaptic elements such as postsynaptic density protein 95 (PSD95) (Scott 2012 ; Sekino et al. 2007). Therefore, different markers of synaptic plasticity were evaluated (Fig. 7). The results obtained showed that LCA induced a significant increase in phospho-cAMP response element-binding (CREB) (Ser 133) in LCA + LPS group compared to LPS (LPS vs LCA + LPS *p < 0,05), a key factor in the maintenance of synaptic changes such as dendritic spines (Ben Zablah et al. 2021).

**Fig. 8:**
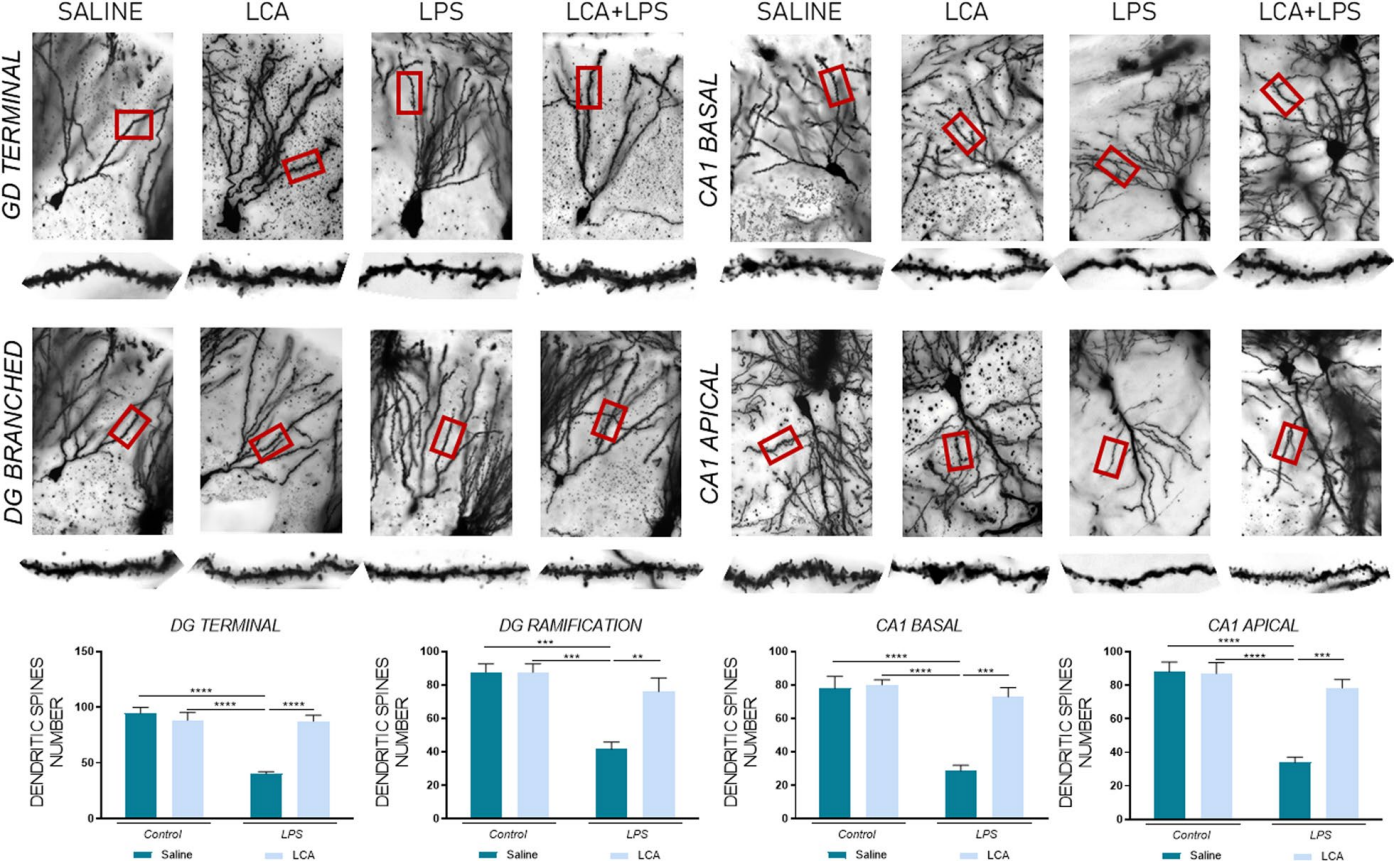
Golgi staining of hippocampal neuron from Dentate Gyrus and CA1 × 63. Representative histogram of dendritic spine accounting. N = 5 independent samples per group, with at least 5 neurons analyzed per sample and zone. Results are expressed as mean ± SEM. Statistical analysis was performed by two-way ANOVA and Tukey´s post-hoc where ** denotes p < 0.01, *** p < 0.001 and **** p < 0.0001

In accordance, animals exposed to LPS showed a significant reduction in dendritic spine number. However, when these animals were previously treated with LCA, dendritic spine preservation was observed in different hippocampal zones, such as the DG and CA1 (Fig. 8) (DG terminal: SAL vs LPS ****p < 0,0001 and LPS vs LCA + LPS ****p < 0,0001, DG ramification SAL vs LPS ***p < 0,001 and LPS vs LCA + LPS **p < 0,01, CA1 BASAL SAL vs LPS ****p < 0,0001 and LPS vs LCA + LPS ***p < 0,001 and CA1 APICAL SAL vs LPS ****p < 0,0001 and LPS vs LCA + LPS ***p < 0,001).

Different synaptic proteins such as PSD95, pro/mature BDNF, drebrin (DBN1) and neuroligin3 (NLG3) were quantified by Western Blot (Fig. 7). These results showed a significant decrease in PSD95, DBN1 and NLG3 in the LPS group compared to saline (SAL vs LPS **p < 0,01 for PSD95 amd***p < 0,001 for DBN1 and NLG3). By contrast, when those animals were previously treated with LCA, no significant differences were observed compared to the control group (ns p > 0,05). Additionally, an increased mature/pro BDNF ratio was observed in LCA + LPS group vs the rest (*p < 0,05).

### LCA improves cognitive decline and depression-like behaviour in lps-treated mice

**Fig. 9:**
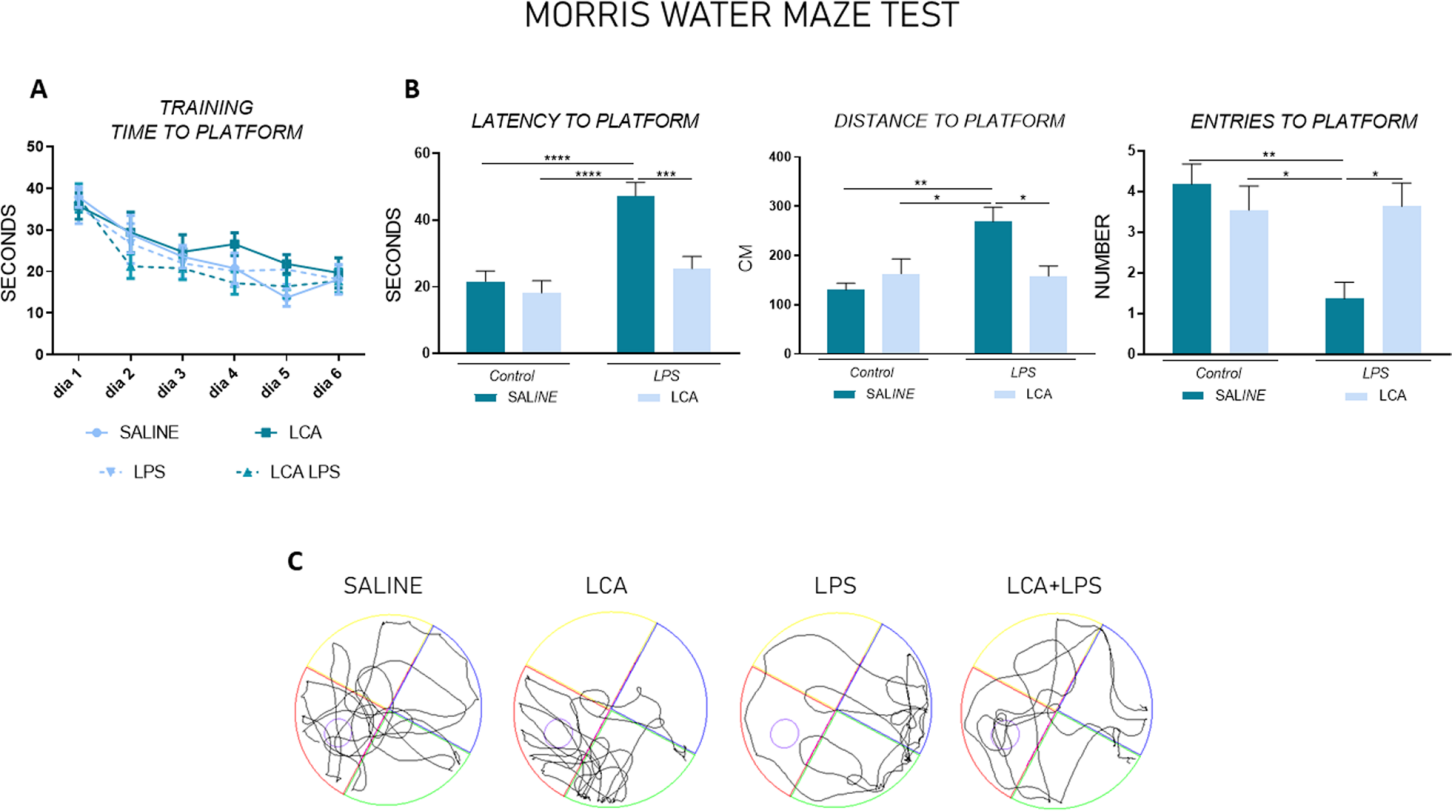
**A**. Representative motion trails of mice on test day. **B** Representative learning curve of mice in the training period. **C.** Representative histogram of latency, distance and entries to platform on the test day (n = 11 independent mice per group). Results are expressed as mean ± SEM. Statistical analysis was performed by two-way ANOVA and Tukey´s post-hoc where * denotes p < 0.05, ** p < 0.01, *** p < 0.001 and **** p < 0.0001

**Fig. 10:**
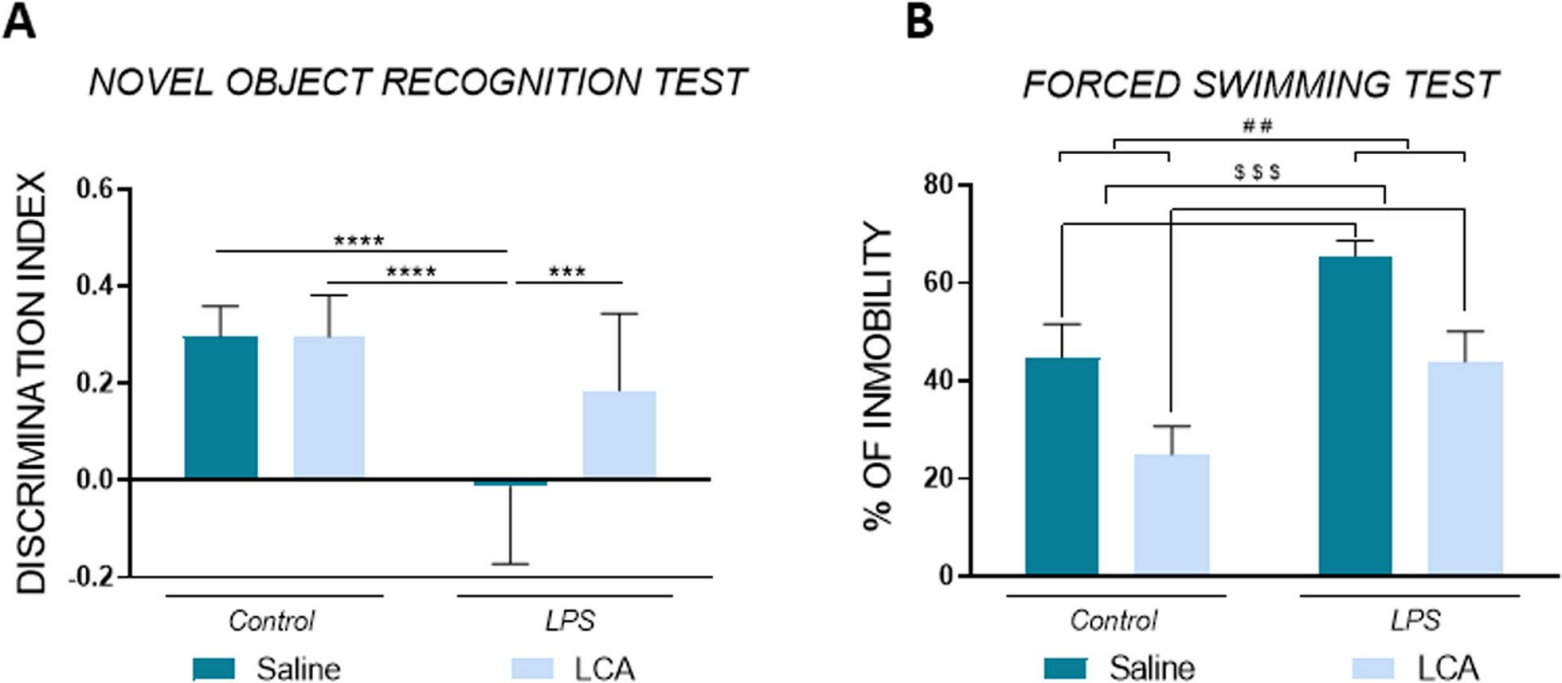
**A**. Representative histogram of Novel Object Recognition Test analysis, expressed as the quantification of the discrimination index (n = 16 independent mice per group). **B.** Representative histogram of Forced Swimming Test, expressed as the % of immobility time (n = 10 independent mice per group). Results are expressed as mean ± SEM. Statistical analysis was performed by two-way ANOVA where $$$ denotes p < 0,0001 compared to non-LCA groups and Tukey´s post-hoc where ** denotes p < 0.01, *** p < 0.001 and **** p < 0.0001

To investigate the neuroprotector effect of LCA against cognitive decline, we conducted MWM and NORT (Figs. 9 and 10, respectively). The data obtained confirmed that a single dose of LPS induces memory loss compared to the other groups in both behavioral tests (SAL vs LPS ****p < 0,0001, **p < 0,01, **p < 0,01 and ****p < 0,0001 in latency to platform, distance to platform and entries in the platform zone of the MWM, as well as, the DI in the NORT, respectively).

Interestingly, when animals were pre-treated with LCA, the results demonstrated an improvement in long-term spatial learning memory in the MWM. This was evidenced by a significant reduction in the time and distance required to reach the platform compared to the LPS group (LPS vs LCA + LPS ***p < 0,001 and *p < 0,05 for time and distance, respectively). Additionally, the number of entries into the platform zone were significantly increased in LCA + LPS group compared to LPS (LPS vs LCA + LPS *p < 0,05). Moreover, long-term recognition memory was also improved in the LCA + LPS group, as indicated by a higher discrimination index compared to LPS group (LPS vs LCA + LPS ***p < 0,001).

Finally, depression-like behavior was studied through FST (Fig. 10). The results demonstrated a significant increase in the percentage of immobility in animals treated with LPS (non-LPS vs LPS **p < 0,01). However, a previous treatment with LCA, clearly reduced the percentage of immobility time in all the animals regardless of LPS exposure (non LCA vs LCA $$$p < 0,001), demonstrating a significant antidepressant effect of LCA.

## Discusion

It is well established that inflammation plays a key role in the development of several neurological disorders, including those characterized by cognitive decline and depression-like symptoms, such as AD (Cunningham and Sanderson 2008). However, inflammation is not the only process involved in these pathologies. It is often accompanied by oxidative stress and metabolic disorders, among others, which are interlinked and gives a high complexity to neurological disorders. In this context, the present study primarily evaluated how LPS administration, as an inductor of inflammation, affects hippocampal molecular mechanisms involved in these pathways through a Taqman Array screening.

Our study demonstrated that a single i.p. dose of LPS could compromise brain neural activity by altering three main targets: 1) enhancement of oxidative stress; 2) brain metabolic alteration and 3) synaptic disturbances. Given that LCA is a multitarget natural compound, its capacity to ameliorate the alterations induced with in vivo LPS administration was evaluated.

Taking all this into account, and supported by several studies, systemic inflammation could be a starting point for the appearance of other pathological processes, collectively contributing to the development of cognitive decline (Kocamer Şahin and Aslan 2024 ). In this context, our study demonstrated that a single LPS administration induces a significant increase of hippocampal glial reactivity, promoting morphological changes in astrocytes and microglia. In contrast, when these animals were pretreated with LCA and then with LPS, no changes in glial morphology were observed compared to controls, demonstrating the anti-inflammatory effect of the compound.

At the molecular level, it is known that LPS promotes proinflammatory cytokine release though TLR4 activation (Lu et al. 2008). Our data corroborated this, showing that LPS administration significantly increases *Tlr4* mRNA expression. Conversely, when mice were treated with LCA, a reduction of *Tlr4* mRNA expression was observed. This confirms the results obtained by Cai et al., who recently demonstrated that the anti-inflammatory effect of LCA was mediated by TLR4 inhibition in an acute lung injury mouse model (Cai et al. 2023).

Conventionally, microglial cells are classified into two subgroups: M1 and M2. M1 is considered a pro-inflammatory status, characterized by an increase in markers such as CD86. In contrast, M2 is defined as an anti-inflammatory stage, characterized by ARG1 expression, among others. In this context, our study demonstrated that LPS administration enhances *Cd86* mRNA in the hippocampus, suggesting the stimulation of M1 phenotype following the LPS exposure. However, in animals treated with LCA, a reduction in *Cd86* mRNA was observed, accompanied by the increase in *Arg1* mRNA expression. These data suggest that while LPS i.p administration promotes the M1 phenotype in the hippocampus, pretreatment with LCA shifts this phenotype to M2.

Additionally, TREM2 has been tightly associated with microglial function during the different stages cognitive decline. Its role, however, has been the subject of considerable debate. Specifically, the induction of TREM2 in AD mice has shown dose-dependent beneficial effects (Tang and Le 2016). Conversely, Zhong et al. demonstrated that soluble TREM2 enhances the expression of pro-inflammatory cytokines in microglia, leading to morphological changes (Merlo et al. 2020; Zhong et al. 2017). In agreement with these latter findings, our study showed a significant increase in *Trem2* expression in animals exposed to LPS, correlating with the previously mentioned microglial morphological changes. However, no changes were observed in control animals or in those mice pretreated with LCA.

As previously mentioned, oxidative stress has been highly related to inflammatory processes and neuronal circuits disruption. In this context, we observed a significant reduction in mRNA expression of antioxidant enzymes such as *Sod1* and *Cat* as well as genes related to cellular respiration, including *Pkm, Pdh1, Ndufv1, Uqcrb, Cycs* and *Cox4i*. This demonstrates that systemic inflammation alters redox balance in the brain, leading to marked release of ROS and subsequent oxidative stress. This process is closely linked to mitochondrial dysfunction, all of which contribute to disturbances in neuronal function (Lee Mosley et al. 2006; Lin and Flint Beal 2006; Rego and Oliveira 2003).

SOD2, the principal mitochondrial antioxidant enzyme (Flynn and Melovn 2013), reduces free radicals and mitigates the damage caused by oxidative stress (Bennett et al. 2009) through the catalyzation of highly reactive O_2_^−^ to less reactive H_2_O_2_ (Kim et al. 2015; Dasuri et al. 2013). Clinical findings have revealed an up-regulation of antioxidant enzymes in the early stages of AD progression, as a compensatory mechanism against elevated levels of oxidative stress observed in pathological stages (Flynn and Melovn 2013; Anantharaman et al. 2006 May).

In this regard, our results did not show modifications in SOD2 protein levels after LPS administration. However, animals treated with LCA exhibited significantly higher levels of this enzyme, likely due to the antioxidant effect of the polyphenol. These results are in the same line with the increase in citrate synthase activity observed in LCA-treated mice, demonstrating proper mitochondrial function (Johnson et al. 2012 ; Cui et al. 2017 ).

Additionally, the presence of ER stress has been reported to impact many mitochondrial functions, such as the transcription of respiratory chain subunits (Koo et al. 2012 ) and ROS accumulation (Cullinan and Diehl 2004 ; Koo et al. 2012; Harding et al. 2003 ). PERK plays an important role by regulating mitochondrial proteostasis and function during ER stress. The dysregulated signaling of this protein has been linked to neurodegenerative diseases (Smith and Mallucci 2016 ; Matus et al. 2011), as high levels of Thr980 phosphorylated PERK have been detected in AD and PD patient’s brains (Stutzbach et al. 2014; Nijholt et al. 2012 ; Scheper and Hoozemans 2015; Rainbolt et al. 2014) (Stutzbach et al. 2014; Nijholt et al. 2012 ; Scheper and Hoozemans 2015). Thus, it has been suggested that the pharmacological inhibition of PERK signaling could confer neuroprotection against multiple neurodegenerative disorders (Radford et al. 2015; Moreno et al. ; Mercado et al. 2018). In this line, our results showed that LPS administration induces PERK phosphorylation, contributing to mitochondrial disturbances, which were reverted with LCA pre-treatment.

Several studies have demonstrated that inefficient glucose utilization and oxidative damage are intimately related. In this context, oxidative modifications in brain mitochondria induce a decrease in glucose metabolism and a consequent reduction in ATP production in the brain, all contributing to synaptic dysfunction (Cunnane et al. 2020 ). In fact, alterations in proteins related to these processes have been observed in patients suffering from mild cognitive impairment to AD (Domenico et al. 2017; Butterfield and Boyd-Kimball 2018). These studies correlate with the results obtained in the present work, which demonstrated the interconnection among inflammation, oxidative stress, metabolism and synaptic disruption.

In accordance, genes related to metabolism, including *Slc2a1, Slc2a2, Prkaa1* and *Gsk3b*, were downregulated after LPS exposure, compromising brain metabolism. Neuronal glucose supply is fundamental to guarantee neuronal homeostasis. In vitro studies have reported that glucose deprivation in hippocampal slices reduces evoked field excitatory post-synaptic potentials and alters long-term potentiation (Harris et al. 2012; Galeffi et al. 2015; Sadgrove et al.). Glucose transporters, specifically GLUT4, play a key role in this process. Therefore, a deficiency of this protein has been related to many neurological disorders, including mild phenotypes of cognitive impairment (Pearson et al.; Pong et al. 2012). Specifically, previous studies have shown that hippocampal inhibition of GLUT4 in rats results in impaired memory acquisition, making it a key regulator of hippocampal memory processing. Moreover, it has been demonstrated that the activation of inflammatory pathways is related to the downregulation of GLUT4 gene expression (Pearson-Leary and McNay 2016 ; Furuya et al. 2013 ; Moraes et al. 2014; Ebersbach-Silva et al. 2018).

In line with this data, the present study revealed a reduction in GLUT4 protein levels in LPS-treated mice. However, when these animals were pretreated with LCA, no differences compared to control group were observed, suggesting glucose homeostasis regulation as a key mechanism underlying LCA’s neuroprotection. The AKT signaling pathway has been described to improve insulin sensitivity and regulating glucose metabolism (Zhou et al. 2021 ). Additionally, it has been described to regulate neuroinflammation, oxidative stress, metabolism and transcriptional regulation (Rai et al. 2019). When AKT is phosphorylated, it induces CREB activation by Ser133 phosphorylation, leading to the transcription of BDNF, among other genes (Brazil et al. 2004 ; Brami-Cherrier et al. 2002 ), a crucial step for the formation of long-term memory and synaptic plasticity (Scott 2012).

However, the function of BDNF is controversial since two isoforms have been detected: pro-BDNF, which mainly binds to the p75 receptor and induces neuronal apoptosis, and mature-BDNF, which primarily binds to TRKB receptor and triggers neuronal development and differentiation, cell survival, long-term potentiation, and synapse plasticity (Teng et al. 2005). Therefore, quantifying mature-BDNF/pro-BDNF ratio is a more discerning indicator than total BDNF levels, as a decreased ratio has been observed in patients displaying early symptoms of neurodegeneration. In accordance, the present data reported a downregulation of P-AKT in cognitive compromised LPS-treated animals. Interestingly, LCA exhibited a positive regulation of this protein levels when compared to LPS group. Regarding P-CREB and BDNF, no changes were observed after LPS administration; however, a significant increase in both protein levels was observed in LCA + LPS mice.

It is widely known that inflammation disrupts the proper synaptic function (Hardiany et al. 2024). In this context, DBN1 is a post-synaptic protein present in excitatory synapses involved in controlling dendritic spine function and morphology. PSD95 is another post-synaptic protein highly abundant in dendritic spines, promoting synapse maturation and facilitating synaptic plasticity. NLG3 has been demonstrated to modulate synapse specialization (Sekino et al. 2007; Hayashi et al. 1996).

Our study has demonstrated that LPS administration reduces the mRNA expression of hippocampal neurexin3 and neuroligin2, as well as PSD95, DBN1 and neuroligin3 protein levels. Moreover, these alterations were reversed in animals pretreated with LCA, leading to dendritic spine maintenance and re-establishment of cognitive function.

In addition, cognitive decline has been associated with depression-like behavior. In fact, some patients suffering from AD manifest depression in the early stages of the pathology, before the development of cognitive deficits. In line with this, our results corroborate the development of depressive symptoms in those animals exposed to LPS (Gouveia et al. 2024),, which aligns with the observed cognitive decline and dendritic spine reduction. By contrast, when these animals were pretreated with LCA, the depressive behavior was reversed.

**Fig. 11:**
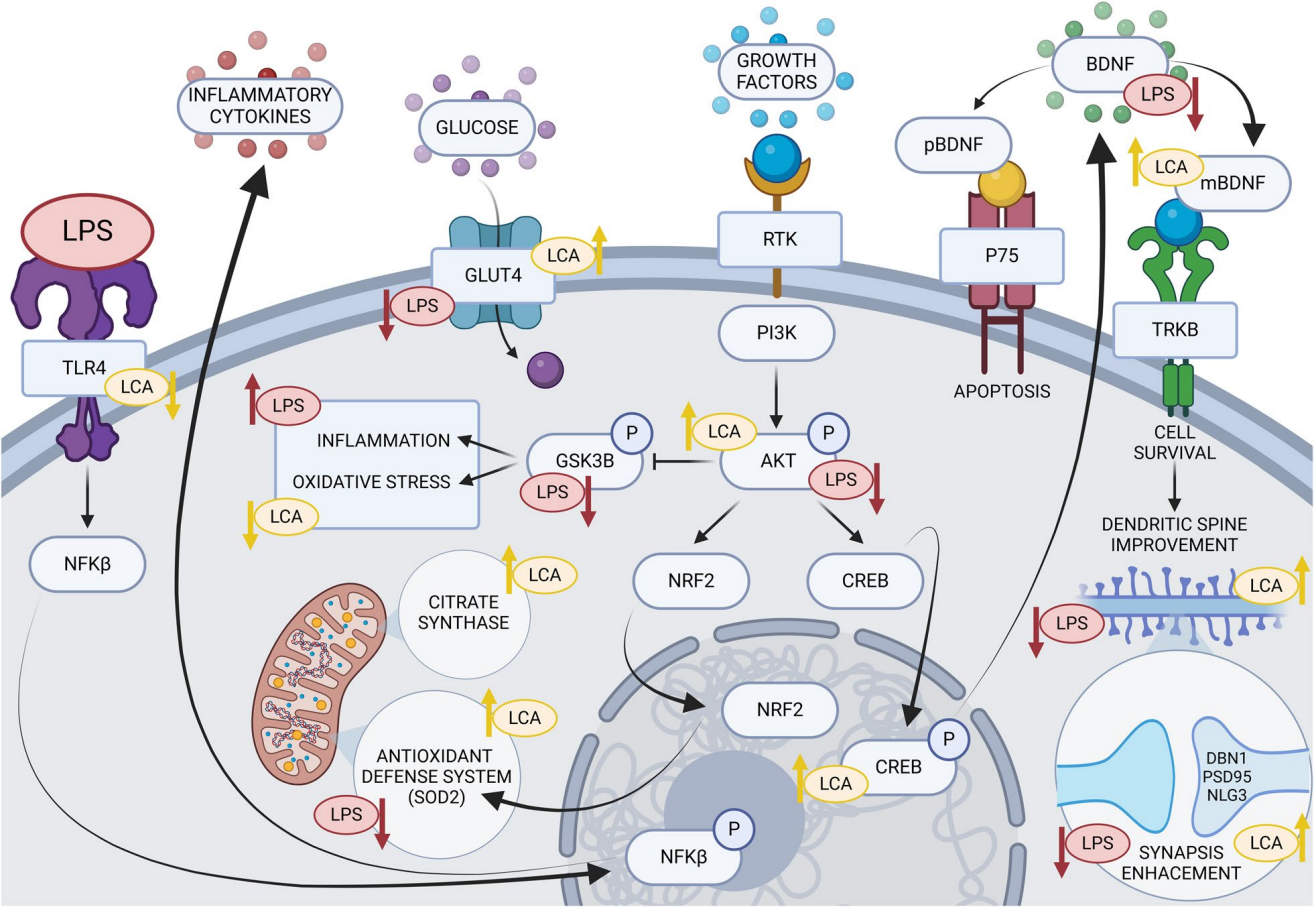
Graphical abstract of LCA neuroprotection mechanism against LPS-induced cognitive decline. Image created with Biorender.com, agreement number YL274HE1CO

In conclusion, according to Fig. 11, the present study demonstrates that LCA can improve cognitive decline and depressive symptoms associated with systemic inflammation by modulating oxidative stress, neuroinflammation and metabolism. Together, these effects contribute to dendritic spine preservation, leading to an improved brain function. Thus, LCA may constitute a multitarget candidate for treating neurological disorders where inflammation plays a significant role.

## Data Availability

No datasets were generated or analysed during the current study.

## Abbreviations

AD: Alzheimer’s disease
ARG1: Arginase 1
BBB: Blood brain barrier
BDNF: Brain-derived neurotrophic factor
CREB: CAMP response element-binding
CNS: Central nervous system
CA1: Cornu Ammonis 1
DG: Dentate gyrus
DEPC: Diethylpyrocarbonate
DI: Discrimination Index
DTT: Dithiothreitol
DBN1: Drebrin
ER: Endoplasmic reticulum
FBS: Fetal bovine serum
FST: Forced Swimming Test
GFAP: Glial fibrillary acidic protein
GLUT4: Glucose transporter type 4
IL-1β: Interleukin-1 beta
IL-6: Interleukin-6
i.p: Intraperitonially
IBA1: Ionized calcium-binding adapter molecule 1
LCA: Licochalcone A
LPS: Lipopolysaccharide
MAPK: Mitogen-activated protein kinases
MWM: Morris Water Maze
NLG3: Neuroligin3
NORT: Novel Object Recognition Test
NF-κβ: Nuclear factor kappa-light-chain-enhancer of activated B cells
O/N: Overnight
PBS: Phosphate buffered saline
PBS-T: Phosphate buffered saline 0.1 M containing 0.5% (v/v) Triton X-100
PERK: Phospho-protein kinase R-like endoplasmic reticulum kinase
PSD95: Postsynaptic density protein 95
AKT: Protein kinase B
ROS: Reactive oxygen species
RT: Room temperature
TLR4: Toll-like receptor 4
TREM2: Triggering receptor expressed on myeloid cells 2
TBS: Tris-buffered saline
TBS-T: Tris-buffered saline with 0.1% (v/v) Tween20
TNF-α: Tumor Necrosis Factor Alpha

## References

[CR1] AlDehlawi H, Jazzar A. The power of licorice (Radix glycyrrhizae) to improve oral health: a comprehensive review of its pharmacological properties and clinical implications. Healthcare. 2023. 10.3390/healthcare11212887. 37958031 10.3390/healthcare11212887 PMC10648065

[CR2] Anantharaman M, Tangpong J, Keller JN, Murphy MP, Markesbery WR, Kiningham KK, et al. β-Amyloid mediated nitration of manganese superoxide dismutase. Am J Pathol. 2006;168(5):1608–18. 16651627 10.2353/ajpath.2006.051223 PMC1606606

[CR3] Ben Zablah Y, Zhang H, Gugustea R, Jia Z. LIM-Kinases in synaptic plasticity, memory, and brain diseases. Cells. 2021;10:2076. 34440848 10.3390/cells10082079 PMC8391678

[CR4] Bennett S, Grant MM, Aldred S. Oxidative stress in vascular dementia and alzheimer’s disease: a common pathology. J Alzheimer’s Dis. 2009;17:245–57. 19221412 10.3233/JAD-2009-1041

[CR5] Bhatia HS, Apweiler M, Sun L, Baron J, Tirkey A, Fiebich BL. Licochalcone a inhibits prostaglandin E2 by targeting the MAPK pathway in LPS activated primary microglia. Molecules. 2023;28(4):1927. 36838914 10.3390/molecules28041927 PMC9965579

[CR6] Borsche M, Pereira SL, Klein C, Grünewald A. Mitochondria and parkinson’s disease: clinical, molecular, and translational aspects. J Parkinsons Dis. 2021;11(1):45–60. 33074190 10.3233/JPD-201981 PMC7990451

[CR7] Brami-Cherrier K, Valjent E, Garcia M, Pagès C, Hipskind RA, Caboche J. Dopamine induces a PI3-Kinase-independent activation of Akt in striatal neurons: a new route to cAMP response element-binding protein phosphorylation. J Neurosci. 2002;22(20):8911–21. 12388598 10.1523/JNEUROSCI.22-20-08911.2002 PMC6757682

[CR8] Brazil DP, Yang ZZ, Hemmings BA. Advances in protein kinase B signalling: AKTion on multiple fronts. Trends Biochem Sci. 2004;29(5):233–42. 15130559 10.1016/j.tibs.2004.03.006

[CR9] Butterfield DA, Boyd-Kimball D. Oxidative stress, amyloid-β peptide, and altered key molecular pathways in the pathogenesis and progression of alzheimer’s disease. J Alzheimer’s Dis. 2018;62(3):1345–67. 29562527 10.3233/JAD-170543 PMC5870019

[CR10] Cai M, Xu Y, Deng B, Chen JB, Chen TF, Zeng KF, et al. Radix Glycyrrhizae extract and licochalcone a exert an anti-inflammatory action by direct suppression of toll like receptor 4. J Ethnopharmacol. 2023;302:115869. 36309116 10.1016/j.jep.2022.115869

[CR11] Chen Y, Qin C, Huang J, Tang X, Liu C, Huang K, et al. The role of astrocytes in oxidative stress of central nervous system: a mixed blessing. In: Cell Proliferation. Hoboken: Blackwell Publishing Ltd; 2020. 10.1111/cpr.12781 PMC7106951 32035016

[CR12] Chunchai T, Chinchapo T, Sripetchwandee J, Thonusin C, Chattipakorn N, Chattipakorn SC. Lipopolysaccharide exacerbates depressive-like behaviors in obese rats through complement C1q-mediated synaptic elimination by microglia. Acta Physiol. 2024. 10.1111/apha.14130. 10.1111/apha.14130 38462756

[CR13] Cui XX, Li X, Dong SY, Guo YJ, Liu T, Wu YC. SIRT3 deacetylated and increased citrate synthase activity in PD model. Biochem Biophys Res Commun. 2017;484(4):767–73. 28161643 10.1016/j.bbrc.2017.01.163

[CR14] Cullinan SB, Diehl JA. PERK-dependent activation of Nrf2 contributes to redox homeostasis and cell survival following endoplasmic reticulum stress. J Biol Chem. 2004;279(19):20108–17. 14978030 10.1074/jbc.M314219200

[CR15] Cunnane SC, Trushina E, Morland C, Prigione A, Casadesus G, Andrews ZB, et al. Brain energy rescue: an emerging therapeutic concept for neurodegenerative disorders of ageing. Nat Rev Drug Discov. 2020;19(9):609–33. 32709961 10.1038/s41573-020-0072-x PMC7948516

[CR16] Cunningham C, Sanderson DJ. Malaise in the water maze: untangling the effects of LPS and IL-1β on learning and memory. Brain, Behavior, Immun. 2008;22:1117–27. 10.1016/j.bbi.2008.05.007 PMC4157220 18640811

[CR17] Dasuri K, Zhang L, Keller JN. Oxidative stress, neurodegeneration, and the balance of protein degradation and protein synthesis. Free Radical Biol Med. 2013;62:170–85. 23000246 10.1016/j.freeradbiomed.2012.09.016

[CR18] Deng N, Qiao M, Li Y, Liang F, Li J, Liu Y. Anticancer effects of licochalcones: a review of the mechanisms. Fronti Pharmacol. 2023. 10.3389/fphar.2023.1074506. 10.3389/fphar.2023.1074506 PMC9900005 36755942

[CR19] Di Domenico F, Barone E, Perluigi M, Butterfield DA. The triangle of death in alzheimer’s disease brain: the aberrant cross-talk among energy metabolism, mammalian target of rapamycin signaling, and protein homeostasis revealed by redox proteomics. Antioxid Redox Signal. 2017;26(8):364–87. 27626216 10.1089/ars.2016.6759

[CR20] Ebersbach-Silva P, Poletto AC, David-Silva A, Seraphim PM, Anhê GF, Passarelli M, et al. Palmitate-induced Slc2a4/GLUT4 downregulation in L6 muscle cells: evidence of inflammatory and endoplasmic reticulum stress involvement. Lipids Health Dis. 2018. 10.1186/s12944-018-0714-8. 29609616 10.1186/s12944-018-0714-8 PMC5879605

[CR21] Flynn JM, Melovn S. SOD2 in mitochondrial dysfunction and neurodegeneration. Free Radical Biol Med. 2013;62:4–12. 23727323 10.1016/j.freeradbiomed.2013.05.027 PMC3811078

[CR22] Furuya DT, Neri EA, Poletto AC, Anhê GF, Freitas HS, Campello RS, et al. Identification of nuclear factor-κB sites in the Slc2a4 gene promoter. Mol Cell Endocrinol. 2013;370(1–2):87–95. 23462193 10.1016/j.mce.2013.01.019

[CR23] Galeffi F, Shetty PK, Sadgrove MP, Turner DA. Age-related metabolic fatigue during low glucose conditions in rat hippocampus. Neurobiol Aging. 2015;36(2):982–92. 25443286 10.1016/j.neurobiolaging.2014.09.016 PMC4443806

[CR24] Gofton TE, Bryan YG. Sepsis-associated encephalopathy. Nat Rev Neurol. 2012;8:8557–66. 10.1038/nrneurol.2012.183 22986430

[CR25] Gong Q, Li W, Ali T, Hu Y, Mou S, Liu Z, et al. eIF4E phosphorylation mediated LPS induced depressive-like behaviors via ameliorated neuroinflammation and dendritic loss. Transl Psychiatry. 2023. 10.1038/s41398-023-02646-5. 37978167 10.1038/s41398-023-02646-5 PMC10656522

[CR26] Gouveia F, Fonseca C, Silva A, Camins A, Teresa Cruz M, Ettcheto M, et al. Intranasal irbesartan reverts cognitive decline and activates the PI3K/AKT pathway in an LPS-induced neuroinflammation mice model. Int Immunopharmacol. 2024;128: 111471. 38199198 10.1016/j.intimp.2023.111471

[CR27] Hardiany NS, Dewi PKK, Dewi S, Tejo BA. Exploration of neuroprotective effect from Coriandrum sativum L. ethanolic seeds extracts on brain of obese rats. Sci Rep. 2024. 10.1038/s41598-024-51221-5. 38182767 10.1038/s41598-024-51221-5 PMC10770154

[CR28] Harding HP, Zhang Y, Zeng H, Novoa I, Lu PD, Calfon M, et al. An integrated stress response regulates amino acid metabolism and resistance to oxidative stress. Mol Cell. 2003;11(3):619–33. 12667446 10.1016/s1097-2765(03)00105-9

[CR29] Harris JJ, Jolivet R, Attwell D. Synaptic energy use and supply. Neuron. 2012;75:762–77. 22958818 10.1016/j.neuron.2012.08.019

[CR30] Hayashi K, Ishikawa R, Ye LH, He XL, Takata K, Kohama K, et al. Modulatory role of drebrin on the cytoskeleton within dendritic spines in the rat cerebral cortex. J Neurosci. 1996. 10.1523/JNEUROSCI.16-22-07161.1996. 8929425 10.1523/JNEUROSCI.16-22-07161.1996 PMC6578938

[CR31] Huang B, Liu J, Ju C, Yang D, Chen G, Xu S, et al. Licochalcone a prevents the loss of dopaminergic neurons by inhibiting microglial activation in lipopolysaccharide (LPS)-induced Parkinson’s disease models. Int J Mol Sci. 2017;18(10):2043. 28937602 10.3390/ijms18102043 PMC5666725

[CR32] Johnson KR, Gagnon LH, Longo-Guess C, Kane KL. Association of a citrate synthase missense mutation with age-related hearing loss in A/J mice. Neurobiol Aging. 2012;33(8):1720–9. 21803452 10.1016/j.neurobiolaging.2011.05.009 PMC3206989

[CR33] Kim GH, Kim JE, Rhie SJ, Yoon S. The role of oxidative stress in neurodegenerative diseases. Exp Neurobiol Korean Soc Neurodegener Dis. 2015;24:325–40. 10.5607/en.2015.24.4.325 PMC4688332 26713080

[CR34] Kocamer Şahin Ş, Aslan E. Inflammation as a neurobiological mechanism of cognitive impairment in psychological stress. J Integr Neurosci. 2024;23(5):101. 38812387 10.31083/j.jin2305101

[CR35] Koo HJ, Piao Y, Pak YK. Endoplasmic reticulum stress impairs insulin signaling through mitochondrial damage in SH-SY5Y cells. Neurosignals. 2012;20(4):265–80. 22378314 10.1159/000333069

[CR36] Lee BH. Neuroprotection: rescue from neuronal death in the brain. Int J Mol Sci. 2021;22:1. 10.3390/ijms22115525 PMC8197180 34073797

[CR37] Lee SY, Chiu YJ, Yang SM, Chen CM, Huang CC, Lee-Chen GJ, et al. Novel synthetic chalcone-coumarin hybrid for Aβ aggregation reduction, antioxidation, and neuroprotection. CNS Neurosci Ther. 2018;24(12):1286–98. 30596401 10.1111/cns.13058 PMC6490010

[CR38] Lee Mosley R, Benner EJ, Kadiu I, Thomas M, Boska MD, Hasan K, et al. Neuroinflammation, oxidative stress and the pathogenesis of parkinson’s disease. Clin Neurosci Res. 2006;6:261. 18060039 10.1016/j.cnr.2006.09.006 PMC1831679

[CR39] Li P, Yu C, Zeng FS, Fu X, Yuan XJ, Wang Q, et al. Licochalcone a attenuates chronic neuropathic pain in rats by inhibiting microglia activation and inflammation. Neurochem Res. 2021;46(5):1112–8. 33555527 10.1007/s11064-021-03244-x

[CR40] Li Z, Wang L, Ren Y, Huang Y, Liu W, Lv Z, et al. Arginase: shedding light on the mechanisms and opportunities in cardiovascular diseases. Cell Death Discov. 2022;8:1. 36209203 10.1038/s41420-022-01200-4 PMC9547100

[CR41] Lin MT, Flint Beal M. Mitochondrial dysfunction and oxidative stress in neurodegenerative diseases. Nature. 2006;443:787–95. 17051205 10.1038/nature05292

[CR42] Lu YC, Yeh WC, Ohashi PS. LPS/TLR4 signal transduction pathway. Cytokine. 2008;42(2):145–51. 18304834 10.1016/j.cyto.2008.01.006

[CR43] Luo Z, Fu C, Li T, Gao Q, Miao D, Xu J, et al. Hypoglycemic Effects of Licochalcone A on the Streptozotocin-Induced Diabetic Mice and Its Mechanism Study. J Agric Food Chem. 2021;69(8):2444–56. 33605141 10.1021/acs.jafc.0c07630

[CR44] Maria Pia GD, Sara F, Mario F, Lorenza S. Biological effects of licochalcones. Mini-Rev Med Chem. 2019;19(8):647–56. 30049263 10.2174/1389557518666180601095420

[CR45] Matsuura S, Nishimoto Y, Endo A, Shiraki H, Suzuki K, Segi-Nishida E. Hippocampal inflammation and gene expression changes in peripheral lipopolysaccharide challenged mice showing sickness and anxiety-like behaviors. Biol Pharm Bull. 2023;46(9):1176–83. 37661396 10.1248/bpb.b22-00729

[CR46] Matus S, Glimcher LH, Hetz C. Protein folding stress in neurodegenerative diseases: a glimpse into the ER. Curr Opin Cell Biol. 2011;23:239–52. 21288706 10.1016/j.ceb.2011.01.003

[CR47] McDonald TS, Lerskiatiphanich T, Woodruff TM, McCombe PA, Lee JD. Potential mechanisms to modify impaired glucose metabolism in neurodegenerative disorders. J Cereb Blood Flow Metab. 2023;43(1):26–43. 36281012 10.1177/0271678X221135061 PMC9875350

[CR48] Medzhitov R, Janeway CA. Decoding the Patterns of Self and Nonself by the Innate Immune System https://www.science.org 10.1126/science.1068883 11951031

[CR49] Mercado G, Castillo V, Soto P, López N, Axten JM, Sardi SP, et al. Targeting PERK signaling with the small molecule GSK2606414 prevents neurodegeneration in a model of Parkinson’s disease. Neurobiol Dis. 2018;112:136–48. 29355603 10.1016/j.nbd.2018.01.004

[CR50] Merlo S, Spampinato SF, Caruso GI, Sortino MA. The ambiguous role of microglia in aβ toxicity: chances for therapeutic intervention. Curr Neuropharmacol. 2020;18(5):446–55. 32003695 10.2174/1570159X18666200131105418 PMC7457435

[CR51] Moraes PA, Yonamine CY, Pinto Junior DC, Esteves JVDC, Machado UF, Mori RC. Insulin acutely triggers transcription of Slc2a4 gene: Participation of the AT-rich E-Box and NFKB-Binding Sites. Life Sci. 2014;114(1):36–44. 25123536 10.1016/j.lfs.2014.07.040

[CR52] Moreno JA, Halliday M, Molloy C, Radford H, Verity N, Axten JM, et al. Oral treatment targeting the unfolded protein response prevents neurodegeneration and clinical disease in prion-infected mice. Neurobiol Dis. 2018. 10.1016/j.nbd.2018.01.004. 10.1126/scitranslmed.3006767 24107777

[CR53] Nijholt DAT, Van Haastert ES, Rozemuller AJM, Scheper W, Hoozemans JJM. The unfolded protein response is associated with early tau pathology in the hippocampus of tauopathies. J Pathol. 2012;226(5):693–702. 22102449 10.1002/path.3969

[CR54] Pearson TS, Akman C, Hinton VJ, Engelstad K, De Vivo DC. Phenotypic spectrum of glucose transporter type 1 deficiency syndrome (Glut1 DS). Curr Neurol Neurosci Rep. 2013. 10.1007/s11910-013-0342-7. 23443458 10.1007/s11910-013-0342-7

[CR55] Pearson-Leary J, McNay EC. Novel roles for the insulin-regulated glucose transporter-4 in hippocampally dependent memory. J Neurosci. 2016;36(47):11851–64. 27881773 10.1523/JNEUROSCI.1700-16.2016 PMC5125244

[CR56] Pong AW, Geary BR, Engelstad KM, Natarajan A, Yang H, De Vivo DC. Glucose transporter type i deficiency syndrome: epilepsy phenotypes and outcomes. Epilepsia. 2012;53(9):1503–10. 22812641 10.1111/j.1528-1167.2012.03592.x

[CR57] Prinz M, Priller J. The role of peripheral immune cells in the CNS in steady state and disease. Nature Neurosci. 2017;20:136–44. 28092660 10.1038/nn.4475

[CR58] Qin L, Wu X, Block ML, Liu Y, Breese GR, Hong JS, et al. Systemic LPS causes chronic neuroinflammation and progressive neurodegeneration. Glia. 2007;55(5):453–62. 17203472 10.1002/glia.20467 PMC2871685

[CR59] Radford H, Moreno JA, Verity N, Halliday M, Mallucci GR. PERK inhibition prevents tau-mediated neurodegeneration in a mouse model of frontotemporal dementia. Acta Neuropathol. 2015;130(5):633–42. 26450683 10.1007/s00401-015-1487-z PMC4612323

[CR60] Rai SN, Dilnashin H, Birla H, Sen SS, Zahra W, Rathore AS, et al. The Role of PI3K/Akt and ERK in Neurodegenerative Disorders. Neurotox Res. 2019;35:775–95. 30707354 10.1007/s12640-019-0003-y

[CR61] Rainbolt TK, Saunders JM, Wiseman RL. Stress-responsive regulation of mitochondria through the ER unfolded protein response. Trends Endocrinol Metab. 2014;25(10):528–37. 25048297 10.1016/j.tem.2014.06.007

[CR62] Rego AC, Oliveira CR. Mitochondrial dysfunction and reactive oxygen species in excitotoxicity and apoptosis: implications for the pathogenesis of neurodegenerative diseases. Neurochem Res. 2003;28(10):1563–74. 14570402 10.1023/a:1025682611389

[CR63] Sadgrove MP, Beaver CJ, Turner DA. Effects of Relative Hypoglycemia on LTP and NADH Imaging in Rat Hippocampal Slices. 10.1016/j.brainres.2007.06.052 PMC2075092 17651706

[CR64] Sanfeliu C, Bartra C, Suñol C, Rodríguez-Farré E. New insights in animal models of neurotoxicity-induced neurodegeneration. Front Neurosci. 2023;17:1248727. 38260026 10.3389/fnins.2023.1248727 PMC10800989

[CR65] Sarkar C, Chaudhary P, Jamaddar S, Janmeda P, Mondal M, Mubarak MS, et al. Redox activity of flavonoids: impact on human health, therapeutics, and chemical safety. chemical research in toxicology. Am Chem Soc. 2022;35:140–62. 10.1021/acs.chemrestox.1c00348 35045245

[CR66] Scheper W, Hoozemans JJM. The unfolded protein response in neurodegenerative diseases: a neuropathological perspective. Acta Neuropathol. 2015;130:315–31. 26210990 10.1007/s00401-015-1462-8 PMC4541706

[CR67] Schindelin J, Arganda-Carreras I, Frise E, Kaynig V, Longair M, Pietzsch T, et al. Fiji: An open-source platform for biological-image analysis. Nat Methods. 2012. 10.1038/nmeth.2019. 22743772 10.1038/nmeth.2019 PMC3855844

[CR68] Scott BR. Cyclic AMP response element-binding protein (CREB) phosphorylation: a mechanistic marker in the development of memory enhancing Alzheimer’s disease therapeutics. Biochem Pharmacol. 2012;83(6):705–14. 22119240 10.1016/j.bcp.2011.11.009

[CR69] Sekino Y, Kojima N, Shirao T. Role of actin cytoskeleton in dendritic spine morphogenesis. Neurochem Int. 2007;51:92–104. 17590478 10.1016/j.neuint.2007.04.029

[CR70] Semmler A, Hermann S, Mormann F, Weberpals M, Paxian SA, Okulla T, et al. Sepsis causes neuroinflammation and concomitant decrease of cerebral metabolism. J Neuroinflammation. 2008;15:5. 10.1186/1742-2094-5-38 PMC2553764 18793399

[CR71] Smith HL, Mallucci GR. The unfolded protein response: Mechanisms and therapy of neurodegeneration. Brain. 2016;139(8):2113–21. 27190028 10.1093/brain/aww101 PMC4958893

[CR72] Smith PLP, Hagberg H, Naylor AS, Mallard C. Neonatal peripheral immune challenge activates microglia and inhibits neurogenesis in the developing murine hippocampus. Dev Neurosci. 2014;36(2):119–31. 24642725 10.1159/000359950

[CR73] Solleiro-Villavicencio H, Rivas-Arancibia S. Effect of chronic oxidative stress on neuroinflammatory response mediated by CD4+T cells in neurodegenerative diseases. Front Cell Neurosci. 2018. 10.3389/fncel.2018.00114. 29755324 10.3389/fncel.2018.00114 PMC5934485

[CR74] Stutzbach LD, Xie SX, Naj AC, Albin R, Gilman S, Lee VMY, et al. The unfolded protein response is activated in disease-affected brain regions in progressive supranuclear palsy and Alzheimer’s disease. Acta Neuropathol Commun. 2014. 10.1186/2051-5960-1-31. 10.1186/2051-5960-1-31 PMC3893579 24252572

[CR75] Sun Y, Zhang H, Liu R, Huang R, Zhang X, Zhou S, et al. Pyrolae herba alleviates cognitive impairment via hippocampal TREM2 signaling modulating neuroinflammation and neurogenesis in lipopolysaccharide-treated mice. J Ethnopharmacol. 2024;30:319. 10.1016/j.jep.2023.117214 37739108

[CR76] Takayuki Irahara M, Norio Sato MP, Kosuke Otake M, Shigenobu Matsumura P, Kazuo Inoue P, Kengo Ishihara P, et al. Alterations in energy substrate metabolism in mice with different degrees of sepsis. J Surg Res. 2018;6(227):44–51. 10.1016/j.jss.2018.01.021 29804861

[CR77] Tang Y, Le W. Differential roles of M1 and M2 Microglia in neurodegenerative diseases. Mol Neurobiol. 2016;53:1181–94. 25598354 10.1007/s12035-014-9070-5

[CR78] Teng HK, Teng KK, Lee R, Wright S, Tevar S, Almeida RD, et al. ProBDNF induces neuronal apoptosis via activation of a receptor complex of p75NTR and sortilin. J Neurosci. 2005;25(22):5455–63. 15930396 10.1523/JNEUROSCI.5123-04.2005 PMC6724992

[CR79] Thomson CA, McColl A, Cavanagh J, Graham GJ. Peripheral inflammation is associated with remote global gene expression changes in the brain. J Neuroinflammation. 2014;8:11. 10.1186/1742-2094-11-73 PMC4022192 24708794

[CR80] Wang X, Quinn PJ. Endotoxins: Lipopolysaccharides of Gram-Negative Bacteria. In 2010. p. 3–25. 10.1007/978-90-481-9078-2_1 20593260

[CR81] Wang S, Fu JL, Hao HF, Jiao YN, Li PP, Han SY. Metabolic reprogramming by traditional Chinese medicine and its role in effective cancer therapy. Pharmacol Res. 2021. 10.1016/j.phrs.2021.105728. 34119622 10.1016/j.phrs.2021.105728

[CR82] Wang D, Yang L, Ding W, Chen Z, Yang X, Jiang Y, et al. Licochalcone a alleviates abnormal glucolipid metabolism and restores energy homeostasis in diet-induced diabetic mice. Phytother Res. 2024;38(1):196–213. 37850242 10.1002/ptr.8044

[CR83] Yankelevitch-Yahav R, Franko M, Huly A, Doron R. The forced swim test as a model of depressive-like behavior. J vis Exp. 2015. 10.3791/52587. 25867960 10.3791/52587 PMC4401172

[CR84] Yating Wu, Zhu J, Liu H, Liu H. Licochalcone A improves the cognitive ability of mice by regulating T- and B-cell proliferation. Aging. 2021;13(6):8895–915. 33714945 10.18632/aging.202704 PMC8034954

[CR85] Yonamine CY, Passarelli M, Suemoto CK, Pasqualucci CA, Jacob-Filho W, Alves VAF, et al. Postmortem brains from subjects with diabetes mellitus display reduced glut4 expression and soma area in hippocampal neurons: potential involvement of inflammation. Cells. 2023. 10.3390/cells12091250. 37174649 10.3390/cells12091250 PMC10177173

[CR86] Zhao J, Zhang M, Zhang H, Wang Y, Chen B, Shao J. Diosmin ameliorates LPS-induced depression-like behaviors in mice: Inhibition of inflammation and oxidative stress in the prefrontal cortex. Brain Res Bull. 2024;1:206. 10.1016/j.brainresbull.2023.110843 38092305

[CR87] Zhong L, Chen XF, Wang T, Wang Z, Liao C, Wang Z, et al. Soluble TREM2 induces inflammatory responses and enhances microglial survival. J Exp Med. 2017;214(3):597–607. 28209725 10.1084/jem.20160844 PMC5339672

[CR88] Zhou Y, Wu R, Cai FF, Zhou WJ, Lu YY, Zhang H, et al. Xiaoyaosan decoction alleviated rat liver fibrosis via the TGFβ/Smad and Akt/FoxO3 signaling pathways based on network pharmacology analysis. J Ethnopharmacol. 2021;264: 113021. 32479885 10.1016/j.jep.2020.113021

